# Comparative analysis of the cellular landscape in mammalian striatum

**DOI:** 10.1038/s41467-026-73305-8

**Published:** 2026-05-25

**Authors:** Gozde Buyukkahraman, Emre Caglayan, Stephen G. Hörpel, Yaqiang Zhang, Ine A. van Tussenbroek, Carlos G. Orozco, Emily Oh, William D. Hopkins, Chet C. Sherwood, Todd F. Roberts, Sonja C. Vernes, Genevieve Konopka

**Affiliations:** 1 Department of Neurobiology, UCLA David Geffen School of Medicine, Los Angeles, CA USA; 2 Department of Neuroscience, Peter O’Donnell Jr. Brain Institute, UT Southwestern Medical Center, Dallas, TX USA; 3 School of Biology, University of St Andrews, St Andrews, UK; 4 Keeling Center for Comparative Medicine and Research, The University of Texas, MD Anderson Cancer Center, Bastrop, TX USA; 5 Department of Anthropology and Center for the Advanced Study of Human Paleobiology, The George Washington University, Washington, DC USA; 6 Present Address: Division of Genetics and Genomics, Department of Pediatrics, Boston Children’s Hospital, Harvard Medical School, Boston, MA USA

**Keywords:** Molecular evolution, Genetics of the nervous system

## Abstract

The dorsal striatum is important for highly specialized functions including movement, learning, and habit formation. However, it is not known if species-specialized behaviors are associated with cellular specializations in the striatum. Here, we compared single-nucleus RNA sequencing (snRNA-seq) data from human, chimpanzee, rhesus macaque, common marmoset, and pale spear-nosed bat caudate (CN) and putamen (Pu) separately as well as mouse caudoputamen (C-Pu), which represents divergence among species spanning approximately 94 million years of evolution. We observed a lower neuron-to-glia ratio in primate striata compared to non-primates, reflecting the allometric scaling of neuron density and relative glia density invariance in larger brains. Among neurons, eccentric spiny projection neurons (eSPNs) - an SPN of unknown function - showed significantly lower proportions in non-primate striata for both CN and Pu. Focusing on the heterogeneity within interneurons, we identified two bat striatal interneuron cell types that are nearly absent in other species: which express *LMO3*, and co-express *FOXP2* and *TSHZ2*. Other striatal interneurons also exhibited differential abundance between primates and non-primates. In summary, we provide a comprehensive snRNA-seq dataset of dorsal striatum, identify two distinct, previously uncharacterized populations of bat interneurons, and uncover fundamental cellular composition differences between primate and non-primate striata.

## Abstract (web summary)

Comparative analyses of striatal cell types across humans, bats and other mammals can reveal how cellular diversity contributes to evolutionary differences in brain function. Here, using snRNA sequencing, the authors show differences in neuron-to-glia ratios, eSPN neuron abundance, and interneuron diversity that point to species-specific features of brain functions.

## Introduction

Comparing the cellular composition of the striatum among humans, primates, and other mammals is compelling because it makes vital functional contributions to behaviors, such as decision making^1^, learning^2^, and (vocal) motor control^3–7^, as well as habit formation^8–10^ and goal-directed behavior^11–13^. The striatum receives most of its input from the cerebral cortex and provides feedback via pallido-thalamic circuits, making the study of striatal cell types relevant to building cell-type-based circuit molecular models^14^. The striatum is also a major interface between cortical regions and subcortical structures in the control of behavior^15^. Not surprisingly, the disruption and dysfunction of striatal circuits lead to many devastating diseases, such as Parkinson’s disease^16^, Huntington’s disease^17–20^, tourette’s syndrome^21,22^ and obsessive-compulsive disorder (OCD)^23–26^, thus making it a key area of study for understanding the pathophysiology of these diseases. Furthermore, an evolutionary perspective is vital for understanding the susceptibility of the human brain to neurodegenerative, neurodevelopmental and psychiatric disorders^27,28^. While much progress has been made in comparing the human brain to a limited number of model organisms, the majority of such studies have examined only a few different regions of comparison, primarily within the cerebral cortex^29–31^. A recent study comparing cortical and striatal interneurons between primates and rodents found a transcriptionally distinct interneuron subtype only in the striatum, with no such observations in the cortex^32^, highlighting the necessity of further cellular resolution comparative investigations of the striatum.

The dorsal striatum is comprised of the caudate nucleus (CN) and putamen (Pu), two morphologically distinct brain regions separated by the internal capsule in carnivores and primates^33,34^. The CN and Pu can be functionally distinguished in human brain imaging studies during certain tasks, such as those involving language or speech^35,36^. Within CN and Pu, GABAergic spiny projection neurons (SPNs) comprise the majority of neurons^37,38^. The SPNs themselves form two major subtypes. The dopamine receptor 1 (DRD1) expressing SPNs (D1 SPNs) form the direct pathway, and project to the globus pallidus internus (GPi) and the substantia nigra pars reticulata (SNr). The dopamine receptor 2 (DRD2) expressing SPNs (D2 SPNs) form the indirect pathway and project to the globus pallidus externus (GPe), which subsequently projects to the subthalamic nucleus (STN)^37,39^^.^ In addition to the two subtypes, a third class of SPNs, eccentric SPNs (eSPNs), has been identified and defined by transcriptomic signatures rather than classical DRD1/2 expression^40,41^. Previous studies have identified and described the function of SPNs that express both D1 and D2 (eSPNs) in rodents^42–46^ and primates^9,47,48^; however, it remains to be determined whether these cells can be characterized as eSPNs.

Interneurons, identified by the neuropeptides that they express, make up the rest of the striatal neurons and regulate the cell signaling, excitability, and spiking timing of SPNs^49,50^. A few studies have begun to compare the cellular composition and cell type expression patterns of the human striatal interneurons with those of other species. For example, Krienen et al. identified a primate-specific striatal interneuron cell type, defined by expression of *TAC3*, which had no homologous molecular counterpart in non-primate striata^32^. Whereas a recent study that expanded the Boreoeutherian mammal species sampled to include ferrets and pigs, showed that the initial classes forming *TAC3* interneurons are conserved across 90 million years of evolution^51^. Using single-nuclear RNA sequencing (snRNA-seq) and spatial transcriptomics, a detailed classification of human striatal interneurons revealed that *PTHLH* and *TAC3* interneurons represent the largest classes of interneuron cell types, with the CN showing a significant abundance of the *PTHLH* subtype compared to the Pu^52^. These studies provide the initial classification of striatal interneurons and reveal their remarkable heterogeneity that has been subjected to evolutionary change. However, a comprehensive comparison of the human striatum to a diverse group of species has yet to be performed.

In this study, to assess the extent of conservation in terms of cell types and gene expression profiles in the dorsal striatum (called “striatum” hereafter) across functionally distinct regions, we generated snRNA-seq datasets of CN and Pu. To make comparisons among primate species, we included data from adult humans, chimpanzees, rhesus macaques, and common marmosets. We found the proportion of neurons was significantly higher in the CN of macaque and marmoset compared to human and chimpanzee, but these differences were not observed in the Pu. To extend these findings to non-primate mammals, we included the adult mouse striatum dataset^32^, and to include a non-primate mammal with distinct CN and Pu structures, we generated CN and Pu snRNA-seq datasets from adult pale spear-nosed bats. We found a strikingly lower neuron-to-glia ratio in primate striata compared to non-primates. By including CN and Pu datasets, we were able to identify cellular features that are conserved across mammalian CN or Pu, as well as features that are unique to pale spear-nosed bats. Together, these findings provide comprehensive cellular and transcriptomic comparisons across mammalian CN and Pu, while simultaneously introducing snRNA-seq datasets of bat and chimpanzee striatum.

## Results

### Identification of striatal cell populations of each species

**Fig. 1:**
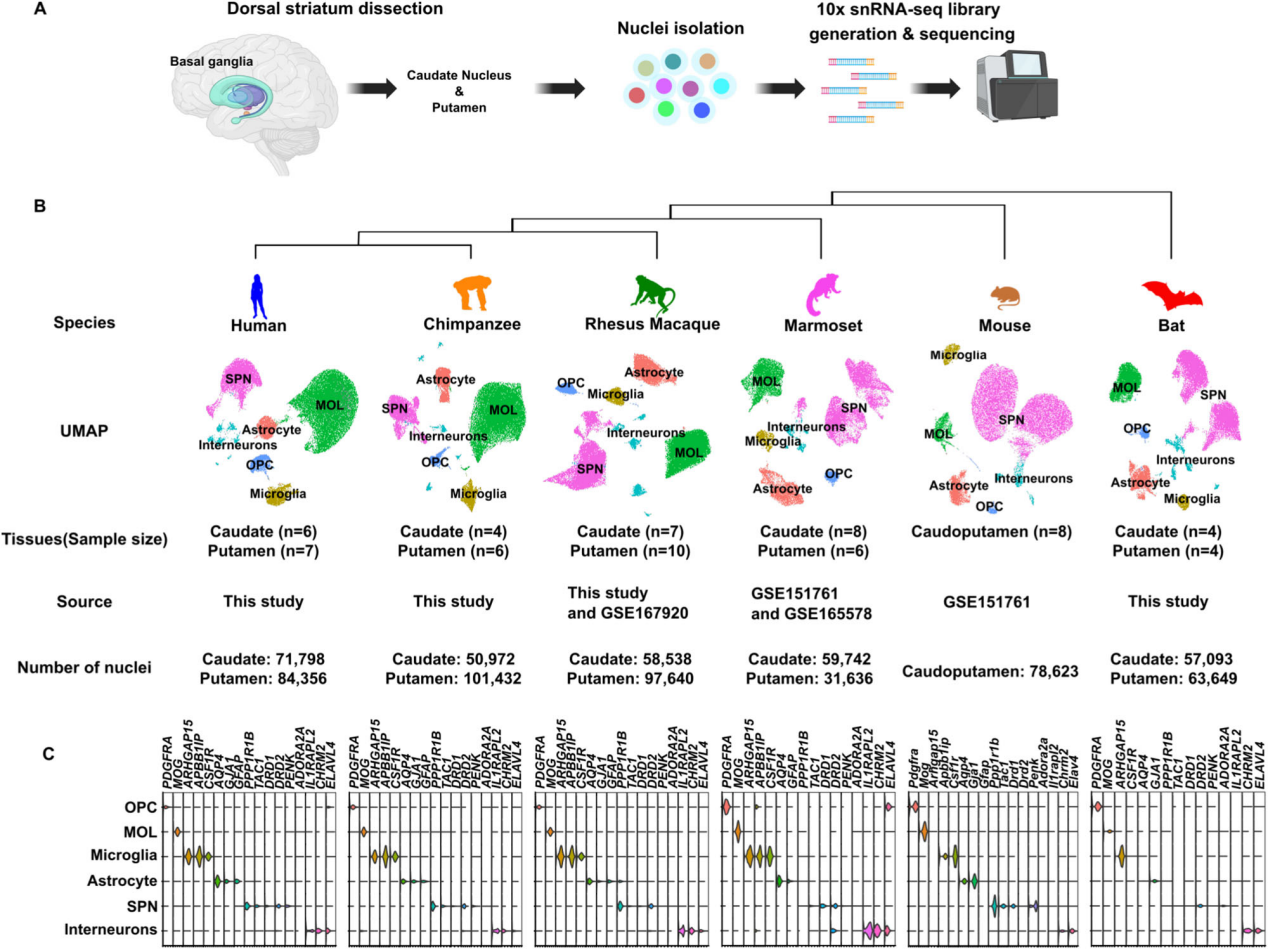
Overview of the snRNA-seq datasets. **A** Schematic illustrating a representative method for profiling the caudate nucleus (CN) and putamen (Pu) via snRNA-seq libraries. **B** Summary of species, major cell type annotations, sample sizes, sources, and the number of nuclei post-filtering for the snRNA-seq datasets analyzed in this study. **C** Violin plots showing the expression of marker genes for major cell types at the single-nucleus level. The shape of the plot represents the density of the nuclei. Source data are provided as a Source data file. Animal silhouettes from PhyloPic (chimpanzee: Caspar K.R.; *Phyllostomus discolor*: Sibaja R.D.; mouse: Miranda-Rottmann S.), used under CC-BY 3.0 (https://creativecommons.org/licenses/by/3.0/); images recolored. **A** Illustrations were created with BioRender.com, Konopka, G. (2026) https://BioRender.com/jle5128.

To understand cellular evolution of the dorsal striatum across diverse mammal species, we generated snRNA-seq data from CN and Pu postmortem brain tissues from adult human (CN *n* = 6 and Pu *n* = 7), chimpanzee (CN *n* = 4 and Pu *n* = 6), rhesus macaque (CN *n* = 4 and Pu *n* = 7), and pale spear-nosed bat (CN *n* = 4 and Pu *n* = 4) (Figs. 1A, S1A, and Supplementary Data 1). In addition to these newly generated datasets, to extend our species comparisons, we included published snRNA-seq datasets from adult rhesus macaque^48^, common marmoset^53,54^, and mouse^32^ (Fig. 1B).

We integrated all datasets from raw reads and implemented consistent quality control to both the new and published datasets (see “Methods”). After stringent quality control, we identified 156,154 cells in human (pre filter  = 185,531), 152,404 cells in chimpanzee (pre filter = 122,274), 156,178 cells in rhesus macaque (pre filter =  163,025), 91,378 cells in marmoset (pre filter = 109,913), 78,623 cells in mouse (pre filter = 91,054), and 120,742 cells in bat (pre filter = 123,362) (Fig. 1 and “Methods”). The mean number of unique molecular identifiers of the samples we sequenced was at or above 1000, comparable to the published datasets (Fig. S1B). To evaluate the quality of the nuclei, we further employed intronic read ratio, a reliable quality control metric for snRNA-seq data^55^, and found that intronic read ratios were consistently high across all samples (Fig. S1C).

We identified major cell types in all species using the conserved cell type markers: *AQP4*, *GJA1,* and *GFAP* for astrocytes, *CSF1R*, *ARHGAP15*, and *APBB1IP* for microglia, *MOG* for mature oligodendrocytes (MOLs), *PDGFRA* for oligodendrocyte progenitor cells (OPCs), *PPP1R1B*, *DRD1, TAC1, PENK, and DRD2* for SPNs, and *IL1RAPL2, CHRM2*, and *ELAVL4* for interneurons (see “Methods,” Figs. 1C and S1D). While we observed similar contributions to cell types across most samples within species, there were striking differences of cell type proportions across species as well as within species between CN and Pu (Fig. S1E, F), which motivated us to examine them further.

### Proportional differences in neurons and glia across species

**Fig. 2:**
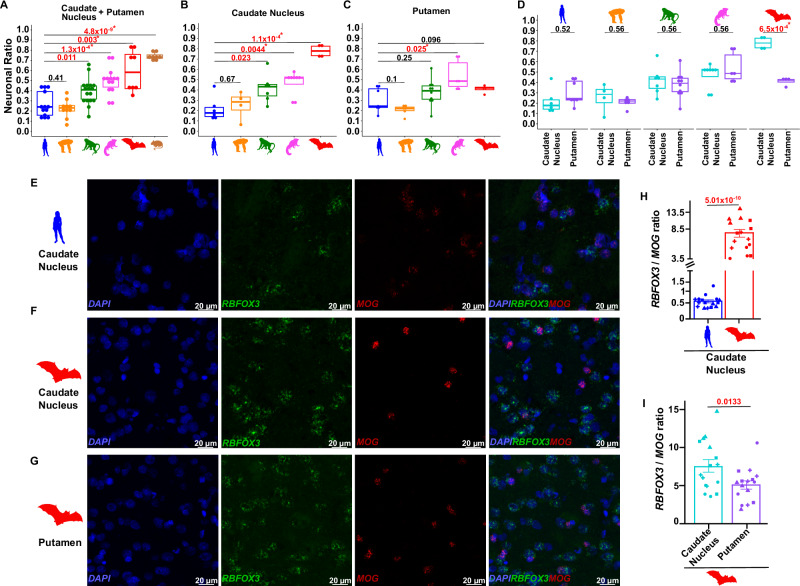
Relative abundance of neurons differs markedly across species. Ratios were calculated by the number of neurons (SPNs and interneurons) divided by the number of total cells (neurons and glia) within each species. We compared each species with human in the following tissue: **A** caudate nucleus (CN) and putamen (Pu) (dorsal striatum) (human, *n* = 13; chimpanzee, *n* = 10; rhesus macaque, *n* = 17; marmoset, *n* = 14; bat, *n* = 8), CN (human, *n* = 6; chimpanzee, *n* = 4; rhesus macaque, *n* = 7; marmoset, *n* = 8; bat, *n* = 4), and Pu (human, *n* = 7; chimpanzee, *n* = 6; rhesus macaque, *n* = 10; marmoset, *n* = 6; bat, *n* = 4). **D** Neuronal proportions were compared between CN and Pu within each species. Two-tailed t-test was used, and nominal *p*-values were multi-test corrected using the Benjamini–Hochberg method to assess statistical significance between the groups (FDR < 0.05 is considered significant and written in red). Additionally, a red asterisk indicates that the comparison is statistically significant in scCODA analysis. Error bars indicate mean ± standard error of the mean individuals per species. Boxplots show the median (center line), interquartile range (box), and whiskers indicating the minimum and maximum values within 1.5× the interquartile range in (**A**–**D**). smFISH validation of **E** human CN, **F** bat CN, and **G** bat Pu. The scale bars represent 20 µm. **H** The ratios of the number of cells co-stained with RBFOX3 and DAPI and the number of cells co-stained with MOG and DAPI in human and bat CN (*n* = 4 with 4 measurements per individual) are shown. smFISH counts were tested using a linear mixed model with species as the fixed effect and individual as the random effect. The *p*-value represents the main effect of species and was determined by a two-sided Wald t-test with Satterthwaite’s approximation. Error bars indicate mean ± standard error of the mean of individuals per species. **I** The ratios of the number of cells co-stained with RBFOX3 and DAPI and the number of cells co-stained with MOG and DAPI in bat CN and Pu (*n* = 4 with 4 measurements per individual) are shown. smFISH counts were tested using a linear mixed model with tissue as the fixed effect and individual as the random effect. The *p*-value represents the main effect of tissue and was determined using a two-sided Wald t-test with Satterthwaite’s approximation. Error bars represent the mean ± standard error of the mean of individuals per tissue. Animal silhouettes from PhyloPic (chimpanzee: Caspar K.R.; *Phyllostomus discolor*: Sibaja R.D.; mouse: Miranda-Rottmann S.), used under CC-BY 3.0 (https://creativecommons.org/licenses/by/3.0/); images recolored.

To determine cell type compositional differences, we first calculated the proportion of neurons (number of neurons divided by the total number of cells, neurons, and glia) in each combined (CN + Pu) dataset and compared these values across species. Human samples had a mean neuron proportion of 26.2%. Interestingly, this ratio increased with increasing evolutionary distance from humans and decreasing brain size, with the exception of chimpanzees (Supplementary Data 2 and Fig. 2A). We tested the statistical significance of the comparisons using both a two-tailed t-test and a more robust proportional analysis of single-cell data (scCODA) that correctly accounts for the assumptions imposed by the nature of the proportional data^56^. While neuronal proportions were similar between human-chimpanzee, macaque and marmoset neuronal proportions were greater than human with either statistical significance or a strong trend (Supplementary Data 2 and Fig. 2A). As an outgroup to the primates, we calculated neuronal proportions in mouse and found that they were significantly higher than humans (two-tailed t-test, FDR = 4.8 × 10^−9^ and scCODA = significant; Supplementary Data 2 and Fig. 2A). Since mouse does not have distinct CN and Pu structures but instead has a caudoputamen (C-Pu) structure, we included another non-primate, bat, where we can distinguish these structures. We calculated the neuronal proportions in bat and found that, similar to the other species, bat has higher levels of neuronal proportions than human (two-tailed t-test, FDR = 0.03 and scCODA = significant; Supplementary Data 2 and Fig. 2A). These results indicate a significantly lower neuron-to-glia ratio in primates, which we interpret to correlate with larger brain size compared to the non-primates in the dataset.

A recent study showed that there are significant differences in striatal interneuron cell type proportions between CN and Pu in the human striatum^52^. However, a comprehensive analysis of the cellular composition, encompassing all cell types in these striatal regions, has not been conducted in detail across multiple species. To investigate potential regional contributions to interspecies variability in neuronal proportions (Fig. 2A), we compared the proportions of neurons in the CN and Pu across species separately. We excluded mice for these comparisons as their C-Pu structure does not morphologically distinguish between CN and Pu. We found similar evolutionarily divergent neuronal proportions in CN but not in Pu, indicating that combined striatal results are primarily driven by the CN samples (Fig. 2B, C). Bat samples were particularly notable as neuronal proportions were ~40% in Pu compared to ~80% in CN (Fig. 2B, C). Comparing neuronal proportions between the CN and Pu within each species, we found that only bat displayed a drastic and significant change with a greater neuronal proportion in CN (two-tailed t-test, FDR = 6.5 × 10^−4^ and scCODA = significant, Fig. 2D). These results indicate that CN and Pu have similar neuronal cell type proportions within most species, but there may be exceptions in some mammals (e.g., bats in this study) with unexplored consequences for neural circuitry and behavior.

We then asked if lower neuronal proportions in human CN were driven by a relative increase in a subtype of glial cells. To answer this question, we calculated the ratio of neuronal to glial cell number in the CN of each species and in the C-Pu of the mouse. We found an overall increase in neuron to glial cell type ratios in other species compared to humans for most glial cell types (Fig. S2A–D), with the exception of astrocytes within primates (Fig. S2C). Using intact tissue sections, we independently verified the human and bat comparison in CN using smFISH for *RBFOX3* (neuronal marker) and *MOG* (oligodendrocyte lineage marker) (*p* = 5.01 × 10^−10^, “Methods”) (Fig. 2E, F, H).

Similarly, we sought to understand whether the neuronal proportion difference between CN and Pu (Fig. 2D) might be driven by proportional changes in glial cell types. We observed that CN had significantly different neuron-to-OPC ratios compared to Pu only in bats (two-tailed t-test, FDR = 2.1 × 10^−3^ and scCODA = significant; Fig. S2E, F) and strong trends in other glial cell types (Fig. S2E–H). To further assess neuron-to-MOL proportions between bat CN and Pu with an orthogonal method, we performed smFISH, which confirmed a significant difference in the neuron-to-MOL ratio (*p* = 0.0133, “Methods”; Fig. 2F–I). Interestingly, the difference between bat CN and bat Pu was especially low in astrocytes, with only a ~1.5-fold increase of neuron/astrocyte in CN compared to at least a 2-fold increase in all other glial cell types (Fig. S2E–H). Given that neuron/astrocyte is also more similar than neuron/ other glial cell types across species (Fig. S2A–D), this might indicate that astrocyte numbers are more likely to scale with neuron numbers across evolution, a plausible outcome given their critical role in neuronal survival both in vivo and in vitro^57,58^. Together, these results show that most neuronal proportional differences across species and CN-Pu are not driven by only one glial cell type, although the number of astrocyte cells scales more consistently with the number of neuronal cells. These findings are congruent with previous observations that neuron density tends to decrease and glial cell density remains invariant with brain size variation in the ventral striatum of primates^59^ and across other brain regions in mammals^60^.

### Non-primates have lower eSPN to SPN proportions

**Fig. 3:**
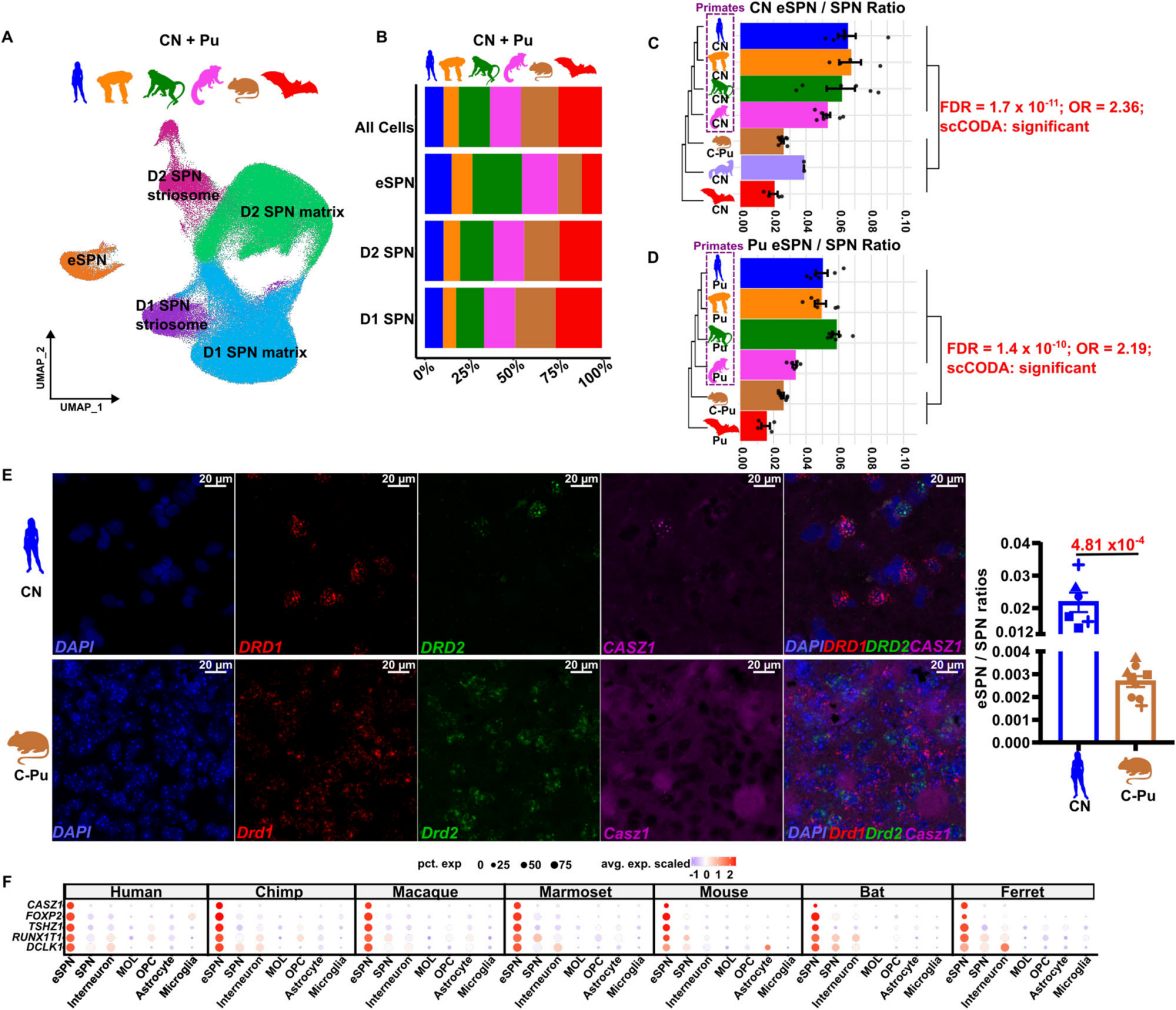
Comparison of spiny projection neuron (SPN) cell types reveals increased eSPN ratios in primates. **A** UMAP of SPNs integrated across human, chimpanzee, rhesus macaque, marmoset, mouse, and bat. **B** Bar plot depicting the proportional compositions of the species across SPN subtypes. **C** Bar plot showing the means of the ratios of the number of eSPNs to the total number of SPNs across the mouse caudoputamen (*n* = 8), and the ferret (*n* = 2), bat (*n* = 4), human (*n* = 6), chimpanzee (*n* = 4), rhesus macaque (*n* = 6), and marmoset caudate nucleus (CN) (*n* = 8). **D** Bar plot showing the means of the ratios of the number of eSPNs to the total number of SPNs across the mouse caudoputamen (*n* = 8), the bat (*n* = 4), human (*n* = 6), chimpanzee (*n* = 6), rhesus macaque (*n* = 7), and marmoset putamen (Pu) (*n* = 6). Two-tailed t-test and scCODA were used to assess statistical significance between the groups. For comparisons where statistical significance was observed, *p*-values are highlighted in red. Source data are provided as a Source data file. **E** smFISH validation of human CN and mouse C-Pu. The scale bars represent 20 µm. The ratio was calculated as follows: the number of cells co-stained with CASZ1, DRD1, and DRD2 and DAPI were divided by the sum of the number of cells co-stained with DRD1 & DAPI and DRD2 & DAPI in human CN (*n* = 2 with 3 measurements per individual). Similarly, for mouse C-Pu, the number of cells co-stained with Casz1, Drd1, and Drd2 and DAPI were divided by the sum of the number of cells co-stained with Drd1 & DAPI and Drd2 & DAPI (*n* = 4 each with at least 2 measurements per individual). smFISH counts were analyzed using a linear mixed model with species as the fixed effect and individual as the random effect. The *p*-value represents the main effect of species and was determined by a two-sided Wald t-test with Satterthwaite’s approximation. **F** Dot plot of eSPN markers across all cell types in human, chimpanzee, rhesus macaque, marmoset, mouse, bat, and ferret. OR odds ratio. Error bars indicate mean ± standard error of the mean of individuals per species. Animal silhouettes from PhyloPic (chimpanzee: Caspar K.R.; *Phyllostomus discolor*: Sibaja R.D.; mouse: Miranda-Rottmann S.), used under CC-BY 3.0 (https://creativecommons.org/licenses/by/3.0/); images recolored.

SPNs comprise the majority of neurons in the striatum, so we identified and annotated the subtypes of SPNs based on the canonical markers *DRD1* and *TAC1* for D1 SPNs, *DRD2* and *PENK* for D2 SPNs, and co-expression of D1 SPN and D2 SPN markers as well as selective expression of *CASZ1* for eSPNs. Besides containing subtypes of SPNs, the dorsal striatum is patterned with functionally and histochemically different structures called the striosome and matrix, and both structures contain all subtypes of SPNs^61,62^. While the functional differences of striosome and matrix compartments are yet to be understood, these compartments are differentially affected in disorders associated with basal ganglia dysfunction^62^. We therefore also distinguished which cells represented striosome or matrix by using canonical markers *SPON1*, *BAXH2*, *SEMA5B*, *KREMEN1*, and *OPRM1* for striosomes, and *CALB1*, *CRYM*, *SGK1*, and *SV2B* for matrix (see “Methods,” Figs. 3A and S3A, B). We found that the distribution of each sample for these cell types was comparable (Fig. S3C, D).

The overall composition of SPNs across species suggested that there may be differences in eSPN proportions, especially in bat and mouse (Fig. 3B). To test this, we calculated the eSPN to SPN ratios and compared non-primates to primates. We found that the relative proportion of eSPNs among all SPNs was significantly less in non-primates (Fig. 3C, D). This difference was significant in both the CN and the Pu (Fig. 3C, D). Additionally, in scCODA^56^, using the D2 SPN cell type as the reference (Fig. S3E), we compared non-primate eSPN proportions to primate eSPN proportions. We found significantly less eSPN to SPN proportions in non-primates compared to primates, thus confirming the t-test statistics results (Fig. 3C, D).

Since the mouse has a C-Pu structure and the bat has morphologically distinct CN and Pu, we asked if having reduced eSPN to SPN proportions is specific to the mouse and bat. To test this, we included another species as an outgroup to primates, where CN and Pu can be separated, a ferret CN dataset^32^ (Fig. S3F, G). We found that high eSPN to SPN proportions were indeed specific to the primates, with non-primates having significantly fewer eSPNs relative to SPNs (FDR = 1.7 × 10^−11^, OR = 2.36, Fig. 3C). We verified these findings using smFISH, where human CN showed significantly higher eSPN to SPN ratios compared to mouse C-Pu (*p* = 4.81 × 10^−4^, “Methods;” and Fig. 3E).

We also examined how similar the eSPNs we identified are to the cells previously identified in the macaque striatum^48^. To achieve this, we performed a correlation analysis of the transcriptional profiles of these cells and found, as previously stated, that they were highly similar (correlation coefficient, *r* = 0.87), sharing most of the expressed genes and their expression levels (Fig. S3H). Additionally, we found that eSPNs mostly expressed striosome markers (Fig. S3B), as previously reported^48^. Since eSPNs are relatively understudied, we wanted to identify what other genetic markers might distinguish eSPNs for future functional studies. We thus performed a Wilcoxon rank sum test to find the genes that are differentially expressed by eSPNs compared to other cell types within each species. We then compared these marker genes across the eSPNs of all species. As expected, human eSPN marker genes showed the greatest overlap with chimpanzee and macaque (Fig. S3I). The second largest overlap of genes was observed within primates (Fig. S3I). We found *CASZ1*, *FOXP2*, *TSHZ1*, *RUNX1T1*, and *DCLK1* genes mark eSPNs consistently in all species (Fig. 3F).

### Identification of interneurons found primarily in the bat

**Fig. 4:**
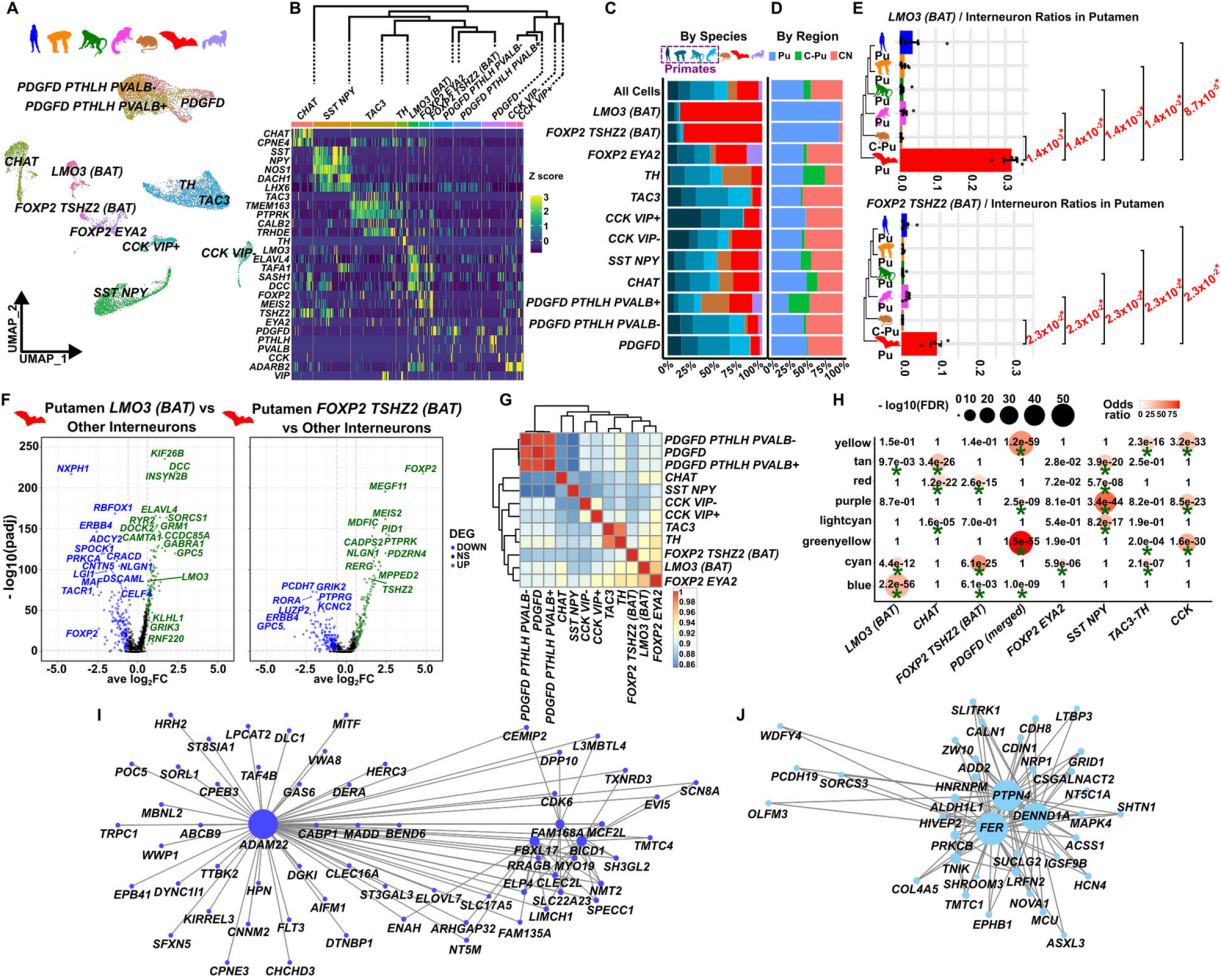
Uncovering striatal interneuron heterogeneity across species reveals previously uncharacterized cell types in bats. **A** UMAP of interneurons integrated across human, chimpanzee, rhesus macaque, marmoset, mouse, bat, and ferret. **B** Heatmap showing the scaled (z-score) gene expression of interneuron cell type markers across the interneuron cell types of all the species. Dendogram shows the hierarchical clustering of the interneuron cell types of all the species. **C** Bar plot depicting the proportional compositions of the species across interneuron subtypes. **D** Bar plot depicting the proportional compositions of the regions across interneuron subtypes. Caudate nucleus (CN), putamen (Pu), and caudoputamen (C-Pu). **E** Bar plots showing the ratio of the number of *LMO3 (BAT)* and *FOXP2 TSHZ2 (BAT)* interneurons to the number of all interneurons across mouse C-Pu (*n* = 8) and bat (*n* = 4), human (*n* = 7), chimpanzee (*n* = 6), rhesus macaque (*n* = 10), and marmoset Pu (*n* = 6). Two-tailed t-test was used, and nominal *p*-values were multi-test corrected using the Benjamini–Hochberg method to assess statistical significance between the groups (FDR < 0.05 is considered significant and written in red). Additionally, a red asterisk indicates that the comparison is statistically significant in scCODA analysis. Error bars indicate mean ± standard error of the mean. **F** Volcano plots showing the upregulated and downregulated genes of *LMO3 (BAT)* and *FOXP2 TSHZ2 (BAT)* interneurons compared to interneurons within bat Pu. Green dots represent significantly upregulated genes, and blue dots represent significantly downregulated genes (Wilcoxon rank-sum test; bonferroni-adjusted *P* < 0.05; log2FC > 0.6 for upregulated genes and log2FC <−0.6 for downregulated genes). **G** Heatmap showing the correlation of normalized gene expressions across interneurons in bat Pu. A Dendrogram depicts the hierarchical clustering of interneurons within bat Pu. **H** Bubble chart depicting enrichment between each gene module and upregulated DEGs (Wilcoxon rank-sum test; log2FC = 0.5, FDR < 0.01) in each interneuron subtype. Green asterisk indicates significance of the overlap in one-sided Fisher’s exact test (FDR < 0.01 and odds ratio > 2). **I**, **J** Representative visualizations of the top 200 connections in the blue module and cyan module. Source data are provided as a Source data file. Animal silhouettes from PhyloPic (chimpanzee: Caspar K.R.; *Phyllostomus discolor*: Sibaja R.D.; mouse: Miranda-Rottmann S.), used under CC-BY 3.0 (https://creativecommons.org/licenses/by/3.0/); images recolored.

Unlike SPNs that project outside the striatum, striatal interneurons largely mediate the local connectivity by regulating the activity of SPNs^50^. Strikingly, striatal interneurons are very heterogeneous despite making up only 5-10% of the striatal neurons^63^. To uncover striatal interneuron heterogeneity across species, we subsetted and integrated interneurons across species and annotated each cluster based on the expression of the following genes: *CHAT*, *SST*, *NPY*, *PVALB*, *TH*, *TAC3*, *CCK*, *VIP*, *PDGFD*, and *PTHLH* (Fig. S4A–C) that resulted in 12 interneuron cell types (Fig. 4A). We then hierarchically clustered the interneuron subtypes based on transcriptional profiles to facilitate understanding of potentially similar cell types (Fig. 4B). The most similar cell types identified include *CCK VIP-* and *CCK VIP+* interneurons, *PDGFD PTHLH PVALB-* and *PDGFD PTHLH PVALB+* interneurons, as well as *FOXP2 EYA2* and *FOXP2 TSHZ2* interneurons.

Surprisingly, we identified two interneuron types in the bat that were either not found or found in very low abundance in the other species (Fig. 4C, E). These interneurons are characterized by the expression of transcription factor *LMO3* and the co-expression of *FOXP2* and *TSHZ2* (Fig. 4B). Notably, both cell types are only present in bat Pu and not found in bat CN (Fig. 4D). Since *FOXP2* is also expressed in SPNs (Fig. S3A), we were curious about the specificity of the *FOXP2 TSHZ2 (BAT)* interneurons. We examined the expression of known SPN marker genes across these cells and found that these interneurons do not express SPN markers (Fig. S4B). We concluded that these cells are therefore interneurons found primarily in bats among the species we have examined.

To quantify this observation, we performed differential cellular abundance tests by using two-tailed t-tests (see “Methods”). We found that both *LMO3 (BAT)* and *FOXP2 TSHZ2 (BAT)* interneurons were significantly enriched in bat Pu samples compared to other species (Figs. 4E and S4C). To test the validity of the t-test statistics, we again used scCODA^56^, with the following cell types as reference due to their high abundance and low dispersion^56^: *PDGFD* interneurons for the CN and C-Pu tissues (Fig. S4D) and *SST NPY* interneurons for the Pu and C-Pu tissues (Fig. S4E). Relative to the proportional changes of the reference *SST NPY* interneurons, both the *LMO3 (BAT)* and the *FOXP2 TSHZ2 (BAT)* interneurons were significantly enriched in almost all comparisons between bat Pu and Pu of other species, except the comparison between bat Pu and marmoset Pu for *FOXP2 TSHZ2 (BAT)* interneurons (Supplementary Data 3 and Fig. 4E). Nonetheless, the scCODA results support the t-test findings (Supplementary Data 3 and Fig. 4E).

The hierarchical clustering performed with all tissues and species showed *LMO3* and *FOXP2 TSHZ2* interneurons were close to *TH* and *TAC3* interneurons (Fig. 4B). These results indicate that in bats, *LMO3* and *FOXP2 TSHZ2* interneurons are cell types that may have been derived from a common thyrotropin-expressing progenitor that also generates *TAC3* and *TH* interneurons. *LMO3* interneurons are 30% of all interneurons in bat Pu, whereas *FOXP2 TSHZ2* interneurons comprise 9% of all interneurons in bat Pu (Supplementary Data 4). Differential gene expression analysis showed that in *LMO3* interneurons, in addition to *LMO3*, the significantly upregulated genes include *ELAVL4*, *DCC*, and *KIF26B* whereas significantly downregulated genes include *RBFOX1*, *ERBB4*, and *NXPH1* (Fig. 4F and Supplementary Data 5). Differential gene expression analysis in *FOXP2 TSHZ2* interneurons showed that, in addition to *FOXP2*, *MEGF11, MEIS2*, and *MDIFC* genes were significantly upregulated, whereas *GRIK2*, *RORA*, and *ERBB4* genes were significantly downregulated compared to other interneurons within bat Pu (Fig. 4F and Supplementary Data 6). To characterize these interneurons better within bat Pu, we performed correlation analysis by generating a correlation matrix from the means of normalized gene expression values for each interneuron cell type (see “Methods”). The correlation analysis showed that *LMO3* interneurons were most similar to *FOXP2 EYA2* and *FOXP2 TSHZ2 (BAT)* interneurons (*r* = 0.96 and 0.93, respectively Fig. 4G). The *FOXP2 EYA2*-*FOXP2 TSHZ2 (BAT)-LMO3 (BAT)* clade was most similar to the *TH*-*TAC3* interneuron clade, indicating that these cells may have similar functions in bat Pu and validating the previous findings with all the species and tissues (*r* = 0.90–0.94, Fig. 4B, G). These results suggest that expanding single-cell RNA sequencing analysis to diverse non-primate species enables the identification of previously uncharacterized interneuron cell types.

To gain more insight into bat-specific interneurons, we performed weighted gene correlation network analysis (WGCNA)^64^ across aggregated and normalized gene expression profiles of all bat interneuron cell types (Methods). We identified blue and cyan modules that were highly enriched for significantly upregulated genes of *LMO3 (BAT)* and *FOXP2 TSHZ2 (BAT)* interneurons, respectively (Fig. 4H–J). To identify active biological processes defining these modules, we performed Gene Ontology (GO) enrichment analysis. This analysis revealed significant enrichment primarily for dendrite- and axon-related processes, which were the only terms consistently identified (Supplementary Data 7). The lack of more specific GO term enrichment likely reflects the limited current understanding of the functional roles of neurons in this brain region.

### Identification of primate and human-specific interneurons

**Fig. 5:**
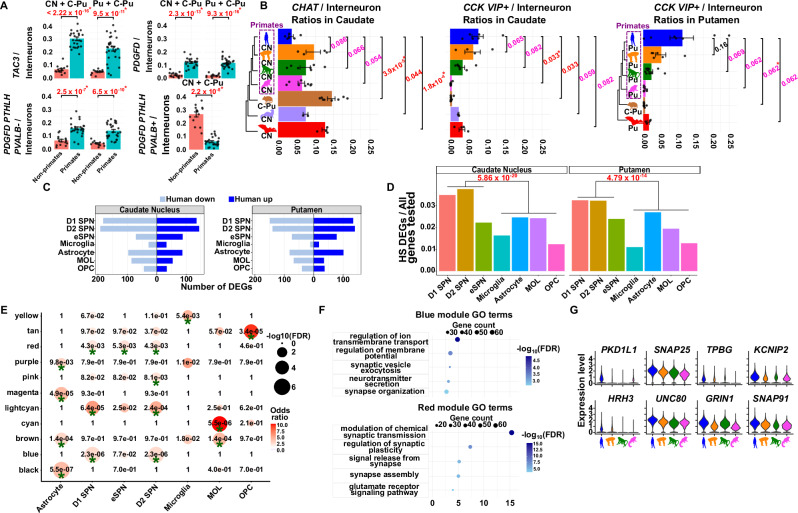
Comparative analysis reveals human-specific changes across striatal interneurons. **A** Bar plot showing the ratio of the number of *TAC3*, *PDGFD*, *PDGFD PTHLH PVALB-*, and *PDGFD PTHLH PVALB+* interneurons to the number of all interneurons across primate and non-primate CN (*n* = 25 and *n* = 14 respectively), Pu and C-Pu (*n* = 29 and *n* = 12 respectively). **B** Bar plots showing the ratio of the number of *CHAT*, *CCK VIP-*, *CCK VIP+* interneurons to the number of all interneurons across mouse caudoputamen (*n* = 8), ferret CN (*n* = 2), and bat (*n* = 4 and *n* = 4), human (*n* = 6 and *n* = 7), chimpanzee (*n* = 4 and *n* = 6), rhesus macaque (*n* = 7 and *n* = 10), and marmoset CN and Pu (*n* = 8 and *n* = 6). Both in **A**, **B**, a two-tailed t-test was used, and nominal *p*-values were multi-test corrected using the Benjamini–Hochberg method to assess statistical significance between the groups (FDR < 0.05 is considered significant and written in red; FDR < 0.1 written in pink). Additionally, a red asterisk indicates that the comparison is statistically significant in scCODA analysis. Error bars indicate mean ± standard error of the mean. **C** Number of human-specific differentially expressed genes (HS DEGs) across cell types. **D** Ratios of HS DEGs to all genes tested for that cell type. Chi-square test was performed to assess significance in ratios between SPNs (D1 SPN, D2 SPN, and eSPN) and glia (MOL, OPC, astrocyte, and microglia). (FDR < 0.05 is considered significant and written in red). E. Bubble plot depicting the enrichment of gene modules with HS DEGs per cell type. Green asterisk indicates significance of the overlap in one-sided Fisher’s exact test (FDR < 0.01 and odds ratio > 2). **F** The top significantly enriched gene ontology terms of the blue and red modules (upper and lower panel, respectively) (FDR < 0.01). **G** Violin plots showing the log-normalized expression of the highly variable HS DEGs across species. CN caudate nucleus, Pu putamen, C-Pu caudoputamen. Source data are provided as a Source data file.

Recent work has found that *TH* and *TAC3* interneurons are transcriptomically similar to each other and concluded that *TH* interneurons are primarily present in mouse, whereas *TAC3* interneurons are primarily present in primates^32,51,65^. In our dataset, in addition to reproducing the primate-specificity of *TAC3* interneurons compared to mouse (32% in CN and 23% in Pu, Supplementary Data 4, FDR < 2.22 × 10^−6^ in CN and FDR = 9.5 ×  10^−11^ in Pu, Fig. 5A), we also identified *TAC3* interneurons in bat (11% in CN, 5% in Pu, Fig. 4C and Supplementary Data 4). Thus, the inclusion of bat, a mammal with a distinct CN and Pu, demonstrates that this cell type is not primate-specific. In our dataset, we also verified that *TH* interneurons are primarily present in mouse samples (16% of all interneurons, Fig. S4C and Supplementary Data 4). However, we found that in addition to *TAC3* interneurons, bat samples contain *TH* interneurons as well (2% in Cu and 3% in Pu, Fig. 4C and Supplementary Data 4), suggesting that *TH* interneurons are not specific to the mouse. In bat Pu, we also found that *TH* and *TAC3* interneurons are correlated in terms of gene expression profiles, corroborating previous findings in other species (Fig. 4G)^32,51,65^. In our dataset, ferret samples did not contain *TH* interneurons; however, *TAC3* interneurons were present in ferret CN (7%, Fig. 4C and Supplementary Data 4) as previously reported^51^. Together, these findings suggest that the *TAC3* and *TH* interneurons are conserved in adult striatum across approximately 94 million years of mammal evolution, as previous studies have also shown^51,66^.

In addition to *TAC3* interneurons, we identified other interneuron subtypes containing a large number of cells: *PDGFD* interneuron classes (Figs. 4A and S4A). In the mouse, it was previously found that *Pvalb* expression does not indicate a discrete class of striatal interneurons but highlights a striatal interneuron class expressing *Pthlh*^67^. In *PVALB* interneurons, in addition to high *PVALB* expression, we also found high *PDGFD* expression; therefore, we labeled these three classes of interneurons as: *PDGFD*, *PDGFD PTHLH PVALB-*, and *PDGFD PTHLH PVALB*+(Figs. 4A, B, and S4A). We found that among these interneuron types, *PDGFD* and *PDGFD PTHLH PVALB-* were primarily present in primates (12% of all interneurons in CN, FDR = 2.3 × 10^−13^, and 12% of all interneurons in Pu, FDR = 9.3 × 10^−16^, and 16% of all interneurons in CN, FDR = 2.5 × 10^−7^, and 15% of all interneurons in Pu, FDR = 6.5 × 10^−10^ respectively; scCODA: significant for all, Figs. 4C, 5A, and Supplementary Data 4) whereas *PDGFD PTHLH PVALB+* was present in CN and C-Pu of non-primates (in mouse C-Pu 31% of all interneurons, in ferret CN 28% of all interneurons, in bat CN 18% all interneurons, Figs. 4C, 5A, and Supplementary Data 4). Interestingly, *PVALB*-expressing *PDGFD PTHLH* interneurons are more abundant in non-primates (bat and ferret CN and mouse C-Pu), whereas the non-*PVALB*-expressing counterpart of the same cell type is more abundant in primates (Fig. 5A). These findings highlight the importance of understanding the striatal *PVALB* activity in future comparative studies (primate vs non-primate) focused on physiology and function.

As our study is powered to interrogate human-specific changes by inclusion of chimpanzee CN and Pu datasets, we then investigated if there are any interneuron subtypes specifically altered in the human lineage in terms of proportional changes. We found a decreasing trend in *CHAT* interneurons in the human striatum compared to other species in our dataset (Fig. 5B). Whereas for both CN and Pu, *CCK VIP+* interneurons showed trends of higher proportions in humans compared to other species (Supplementary Data 8, 9 and Fig. 5B). Our findings highlight species-specific differences in the composition of striatal interneuron cell types, with human samples showing distinct patterns compared to non-primates and marmoset samples.

### Differential gene expression analysis reveals stronger human specific change in SPNs than glia

Recent studies found species-specific cell types in the striatum but not in cortex^32^. Expanding on this trend, we identified two previously uncharacterized distinct interneuron subtypes in *P. discolor* (Fig. 4). Although we did not observe differences in cell-type abundances among primates, we asked whether transcriptomic differences were present among primate striatal cell types. Therefore, we performed differential gene expression (DGE) analysis within primates across both tissues and all cell types: D1 SPN, D2 SPN, eSPN, interneurons, MOL, OPC, astrocyte, and microglia. We further identified the human-specific differentially expressed genes (HS DEGs) for each cell type (“Methods”). We found that, overall, there were more HS up and downregulated genes among D1 and D2 SPNs compared to other cell types (Fig. 5C). To statistically test this, we performed chi-square test for the proportions of HS DEGs to all genes tested within that cell type between neurons and glia (Methods) which revealed significantly more HS DEGs in SPNs than glia regardless of the brain regions (Fig. 5D).

To gain more insight into human-specific changes in SPNs, we performed WGCNA and found the gene modules for each cell type in humans (“Methods”). We uncovered that the blue and red modules represent the genes that are highly correlated with the previously identified HS upregulated DEGs in SPNs (Fig. 5E). The GO-term enrichment analysis of these modules (“Methods”) revealed neurotransmitter related activity in blue module genes whereas it revealed synapse related processes for red module genes (Supplementary Data 10 and Fig. 5F). A closer look on these modules yielded several important genes implicated in human disease: genetic ablation of trophoblast glycoprotein, *Tpbg*, in mice showed motor impairments reminiscent of Parkinson’s disease symptoms^68,69^; *Hrh3*, is a histamine receptor involved in pathophysiology of a mouse model of a tic disease^70,71^; mutations of *UNC80* are involved in regulation of neuron electric signals, and cause dyskinesia, developmental delay and/or severe intellectual disability^72,73^ (Fig. 5G). Overall, these results indicate an excess of human-specific gene regulation in SPNs compared to other cell types in our dataset and demonstrate the importance of these genes in brain function and disease risk.

## Discussion

Cell-type-specific genomics of subcortical brain regions remains underexplored, with a limited availability of single-cell-resolution high-throughput datasets for these areas. The dorsal striatum is an integral part of the forebrain and is involved in decision making, motor control, and reward processing^74^. In this study, we generated and analyzed a comprehensive snRNA-seq dataset, encompassing the CN and Pu across four primates—human, chimpanzee, rhesus macaque, and common marmoset—and three non-primate species: pale spear-nosed bat, ferret, and mouse C-Pu (Fig. 1). By including bat CN and Pu, we were able to identify two previously uncharacterized interneuron cell types, *LMO3* and *FOXP2 TSHZ2* in the bat Pu, their functional roles will be explored in future studies. These findings help underscore the unique characteristics of non-primate striatal anatomy and physiology (Fig. 4A–E). Additionally, we identified significant differences in primates, including lower neuron-to-glia ratios reflecting the allometric scaling of the neuron density and relative glial cell density invariance across different brain sizes, as previously shown in ventral striatum^59^ and other regions^60^ (Fig. 2) and higher eSPN-to-all SPN ratios in comparison to non-primate striata (Fig. 3). Furthermore, we could also identify tissue-specific differences. In bat CN, we found that there is a higher neuron-to-glia ratio than in bat Pu. The ratios of *PDGFD PTHLH PVALB+* interneurons to all interneurons were higher in non-primate CN samples compared to primate CN samples; however, bat Pu showed similar proportions to Pu of primates (Supplementary Data 4). We further found that SPNs, the principal neuronal population of the striatum, exhibit the strongest human-specific transcriptional divergence among all cell types, with enriched changes in genes related to synaptic function and neurotransmitter signaling, many of which are also implicated in neurological disease, underscoring their potential importance in human-specific brain function and vulnerability. Our work parallels previous work on the striatum by highlighting the conservation of important cell types and transcriptomic profiles across approximately 94 million years of mammal evolution^32,40,48,67,75^. Together, our results suggest that the species-specific and tissue-specific specializations of neurons, interneuron cell types and cell type abundances could offer insights into species-specific behavioral differences and disease vulnerability.

Although they are few in number, interneurons not only regulate the basal ganglia circuitry by integrating incoming signals from various brain regions and influencing the activity of SPNs to modulate output signals, but they are also involved in changes related to plasticity and adaptation in disease conditions^49,76,77^. Previous studies reported differences in striatal interneuron number, location and physiological features across various species^32,67,78–81^. The predominance of these previously uncharacterized interneuron cell types in the pale spear-nosed bat highlights distinct, species-specific features of the striatum. Hierarchical clustering analysis indicates that the *LMO3*- and *FOXP2/TSHZ2*-expressing interneurons share substantial transcriptomic similarity with *TAC3-TH* interneurons, suggesting a potential origin from the same progenitor class (Fig. 4B). Previous studies have shown the extensive heterogeneity in tyrosine hydroxylase (TH) expressing cells of adult mice^82,83^. In embryonic mouse stem cells, *Lmo3*, together with *Pou3f4*, was found to be necessary for the differentiation of cortical inhibitory neurons^84^. Additionally, during both human and mouse dopaminergic neuron development, it was found that *LMO3* was co-expressed by a subset of TH cells^85^. However, further studies are needed to understand the function of *LMO3* in bat adult striatal interneurons. Previous studies suggest that FOXP2 has a role in striatal function and speech related motor skills^86–89^. In adult mice, *Tshz2* was shown as a marker for a cell type found in the prefrontal cortex^90^ as well as amygdala excitatory neurons^91^; however, the function of *TSHZ2* has yet to be characterized in adult striatal interneurons and in other species. Additionally, it is not yet understood whether these interneurons emerge from the same progenitor cell as *TH*-*TAC3* interneurons. Future electrophysiological and imaging studies can delineate the functions of these interneuron cell types in bat Pu.

With the advancement of single-cell technologies, cell type innovation has been discovered across species in brain^29,32,40^ and in other organs^92,93^. Expanding evolutionary distance by incorporating a diverse range of species and tissues should uncover alterations in cell types and proportions and enhance our understanding of species-specific variation in cellular identity and behavior across evolutionary lineages. As an example of including non-model species to investigate cell type function, in a recent study performed on oldfield mice (*Peromyscus polionotus*), a link between a recently evolved cell type and parental behavior was found^94^. While we have observed strong transcriptional conservation of the majority of cell types across species in either the caudate or putamen, as might be expected, a caveat to our study is that we cannot be completely certain that we are comparing functionally homologous regions. Future studies that manipulate cell types in model systems, such as the marmoset or ferret, may ultimately validate the direct cell type comparisons. In summary, our findings corroborate previous snRNA-seq studies on the striatum and highlight the critical importance of incorporating a broad range of species when identifying previously uncharacterized cell types and investigating variations in cellular abundance. Future studies examining the functional roles of these previously uncharacterized cell types will deepen our understanding of subcortical brain regions and may provide insight into species-specific behaviors.

## Methods

### Tissue collection

#### Human and non-human primates

All human tissue was obtained from NIH NeuroBioBank, and while rostral samples were requested, we cannot independently verify the precise tissue dissection location. Chimpanzee tissues were obtained from individuals previously housed at either the Emory National Primate Research Center or the Michale E. Keeling Center for Comparative Medicine and Research. Macaque tissues were obtained from the Michale E. Keeling Center for Comparative Medicine and Research. Caudate and putamen were dissected from frozen postmortem tissue slabs (Supplementary Data 1). We sampled nonhuman primate brains from coronal slabs at the level of the rostral striatum, where the caudate and putamen are both prominent and the internal capsule has begun to separate them. This region is anterior to the appearance of the globus pallidus and thalamus.

#### Bats

The adult *Phyllostomus discolor* bats originated from a breeding colony situated in the Department Biology II of the Ludwig-Maximilian University of Munich or in St Mary’s Animal Unit of the University of St Andrews. Animals were kept under semi-natural conditions (12 h day/12 h night cycle, 65–70% relative humidity, 28 °C) with access to food and water ad libitum. All the experiments complied with the principles of laboratory animal care and the regulations of the current version of the German Law on Animal Protection and the Animal Scientific Procedures Act (1986) under Home Office (UK) supervision. The number of animals used in terminal experiments was reported to the Munich veterinary office and the Home Office (UK).

In order to collect the samples used in the single-nuclear RNA sequencing experiments, the animals were euthanized by an intraperitoneally applied lethal dose of pentobarbital (0.16 mg/g bodyweight) followed by decapitation. The brain was extracted, embedded in 3% low melting point agarose and sliced in 500 µm coronal slices on a vibratome (Precisionary Compresstome® VF-200 and Leica VT1200S) in ice-cold buffer solution (3 mM KCl, 3 mM MgCl_2_·6H_2_O, 23 mM NaHCO_3_, 1.2 mM NaH_2_PO_4_·H_2_O, 210 mM sucrose, 11 mM glucose). Tissue punches (1.5 mm diameter, KAI Europe GmbH) were taken from the slices and were snap-frozen in liquid nitrogen.

To harvest tissue used in immunofluorescence experiments, the animals were sedated with MMF (medetomidine, midazolam and fentanyl at a dosage of 0.4, 4.0, and 0.04 µg/g body weight, respectively), euthanised with an intraperitoneal injection of pentobarbital (0.16 mg/g bodyweight) and perfused transcardially with approximately 200 ml of phosphate-buffered saline (PBS; pH 7.4; 0.9% NaCl and 1% heparin 25,000 IU) for 10 minutes, followed by approximately 200 ml of 4% paraformaldehyde in PBS over ~15–20 min. Samples were embedded in OCT (optimal cutting temperature) compound and frozen by isopentane bath. The samples are left in dry ice and stored at −80 °C until sliced. The brains were sliced from a coronal position, using a Leica CM1860 cryostat, 15 microns thick and placed on positively charged glass slides (Superfrost Plus Gold). The slides were then stored at −80 °C until used. No sex-based analyses were performed, as this study focuses on species-level comparisons of gene expression and cellular composition rather than sex-specific effects.

### Single-nucleus RNA library generation and sequencing

#### Bat samples

Nuclei for snRNA-seq were isolated from bat putamen and caudate brain tissue. Briefly, the tissue was homogenized in 500 µL of ice-cold Nuclei EZ lysis buffer (EZ PREP NUC-101, Sigma) using a pre-chilled glass Dounce homogenizer. Debris was removed via density gradient centrifugation using Nuclei PURE 2 M sucrose cushion solution and Nuclei PURE sucrose cushion buffer (Nuclei PURE prep isolation kit, no. NUC201-1KT, Sigma Aldrich). These two solutions were mixed in a 9:1 ratio, and 500 µL of the sucrose solution was added to a 2-ml Eppendorf tube. The homogenized nuclei suspension was mixed with 900 µL of sucrose cushion solution by pipetting 10 times, resulting in a 1400 µL volume, which was carefully layered over the sucrose buffer without mixing. The samples were centrifuged at 13,000 × *g* for 45 min at 4 °C, and all but 100 µL of supernatant was discarded. Nuclei were resuspended in 300 µL of nucleus suspension buffer (NSB), consisting of 1× PBS, 2% BSA (no. AM2618, Thermo Fisher Scientific), and 0.4 U µL^−1^ RNAse inhibitor (no. AM2694, Thermo Fisher Scientific). Samples were then centrifuged at 550 × *g* for 5 min at 4 °C, and all but 50 µL of supernatant was discarded. The nuclear pellet was resuspended in NSB and filtered through a 40-μm Flowmi cell strainer (Bel-Art Products, #H13680-0040) into a new tube. The nuclei concentration was measured using Hoechst and decreased to 1000 nuclei per µL with NSB if necessary.

Droplet-based snRNA-seq libraries were prepared using the Chromium Single Cell 3′ v3.1 (10x Genomics) kit, following the manufacturer's protocol. Libraries were sequenced on an Illumina NovaSeq 6000.

#### Human, chimpanzee, and rhesus macaque samples

Nuclei for snRNA-seq were isolated from the putamen and caudate nuclei of human, chimpanzee, and rhesus macaque tissues. Briefly, the tissue was homogenized in 750 µL of ice-cold NP40 lysis buffer (10 mM Tris-HCl, pH 7.4, 10 mM NaCl, 3 mM MgCl₂, 0.1% Nonidet P40 Substitute, 1 mM DTT, 0.2 U/µL RNAse inhibitor) using a pre-chilled glass Dounce homogenizer. An additional 750 µL of ice-cold NP40 lysis buffer was added, and the mixture was pipette-mixed during a 5-min incubation on ice. Nuclei were centrifuged at 500 × *g* for 5 min at 4 °C, resuspended in 1 mL of ice-cold lysis buffer, and incubated on ice for 5 min with gentle pipette mixing throughout. The nuclei were then centrifuged again at 500 × *g* for 5 min at 4 °C, and all but 50 µL of supernatant was discarded. Next, 500 µL of nucleus suspension buffer (NSB) containing 1× PBS, 1% Ultrapure BSA (no. AM2618, Thermo Fisher Scientific), and 0.2 U/µL RNAse inhibitor (no. AM2694, Thermo Fisher Scientific) was added, and the sample was incubated for 5 min on ice without mixing. The pellet was resuspended, centrifuged again at 500 × *g* for 5 min at 4 °C, and all but 50 µL of supernatant was discarded. The nuclei were then resuspended in 500 µL of NSB and filtered through a 70-μm Flowmi cell strainer (no. H13680-0070, Bel-Art) into a 1.5 mL tube.

Debris was removed using density gradient centrifugation with Nuclei PURE 2 M sucrose cushion solution and Nuclei PURE sucrose cushion buffer (Nuclei PURE prep isolation kit, no. NUC201-1KT, Sigma Aldrich). The solutions were mixed in a 9:1 ratio, and 500 µL of the resulting sucrose solution was added to a 2-ml Eppendorf tube. A 900 µL volume of sucrose buffer was added to 500 µL of isolated nuclei in NSB, and the resulting 1400 µL suspension was layered on top of the sucrose buffer. The gradient was centrifuged at 13,000 × *g* for 45 min at 4 °C. After centrifugation, all but 100 µL of supernatant was removed, and the nuclei were resuspended in 400 µL of NSB, centrifuged at 500 × *g* for 5 min at 4 °C, and all but 50 µL of supernatant was discarded. The nuclei were then resuspended in 200 µL of NSB and filtered through a 40-μm Flowmi cell strainer (no. H13680-0040, Bel-Art). The concentration of nuclei was determined using 0.4% trypan blue (no. 15250061, Thermo Fisher Scientific) and adjusted to a final concentration of 1000 nuclei per µL with NSB.

Droplet-based snRNA-seq libraries were prepared using Chromium Single Cell 3′ v3.1 (1000121, 10x Genomics) according to the manufacturer’s protocol. Libraries were sequenced on an Illumina NovaSeq 6000.

### Single-nucleus RNA sequencing dataset alignment and quality control

We obtained raw snRNA-seq data for human, chimpanzee, macaque, and pale spear-nosed bat libraries from the McDermott Sequencing Core at UT Southwestern in binary base call (BCL) files. The BCL files were demultiplexed using CellRanger bcl2fastq v2.20.0 and CellRanger mkfastq (10x Genomics Cell Ranger 6.0.0) with default parameters to generate FASTQ files. For the previously published rhesus macaque^48^, common marmoset^53,54^, mouse^32^, and ferret^32^ datasets, the FASTQ files were downloaded, and preprocessing was carried out similarly to our own datasets.

Since any non-human primate has a less accurate GTF file than a human GTF file, we converted the positions of every non-human primate to the human annotation file using liftoff (v1)^95^. We used the following genomes: Human (*Homo Sapiens*) hg38 p13, Chimpanzee (*Pan troglodytes*) panTro5, macaque (*Macaca mulatta*) rheMac10, common marmoset (*Callithrix jacchus*) caljac3, mouse (*Mus musculus*) mm10, pale spear-nosed bat (*Phyllostomus discolor*) GCF_004126475.2_mPhyDis1.pri.v3, and ferret (*Mustela putorius furo*) GCF_011764305.1_ASM1176430v1.1. The index for each genome file was generated with CellRanger mkref (10x Genomics Cell Ranger 6.0.0). The FASTQ files were aligned to their corresponding genomes using CellRanger count (10x Genomics Cell Ranger 6.0.0).

To remove ambient RNA contamination, CellBender^96^ was used on the raw count matrices. With these filtered gene-cell matrices, the Seurat objects were generated. To assess the health of the nuclei, we used intronic read ratios^55^. Briefly, we counted the number of reads that are mapped to introns and divided them to the total number of mapped reads within a cell. During clustering, we removed the clusters with exceptionally low mean intronic read ratios (<0.5). In our dataset, the healthy nuclei had intronic read ratios ~≥0.7 (Fig. S1B).

### Single-nucleus RNA sequencing, cell type annotation, and cellular compositional abundance analysis

For each sample (individual), we normalized gene counts using log normalization, found variable genes necessary to generate principal components using FindVariableFeatures^97^. We then selected the top 2000 genes that are variable across samples using SelectIntegrationFeatures with default parameters^97^. We generated z-scores of these genes using ScaleData then integrated samples within each species using FindIntegrationAnchors with normalization.method = “LogNormalize” and reduction method rcpa and IntegrateData with k.weight = 30, normalization.method = “LogNormalize”^97^. We then generated principal components using RunPCA with default parameters and clustered cells using FindNeighbors with SNN and FindClusters with the Louvain algorithm (resolution 1) and visualized using RunUMAP (dims = 1:20 and reduction = “pca”) and DimPlot^97^. We removed clusters with low intronic read ratios (<0.5), we also removed clusters with an unusually high number of detected genes, accompanied with a high level of expression of at least two typically distinct marker genes, as potential nuclear doublets. In addition to doublets, we also removed non-cells (empty), endothelial cells, and pericytes (*FLT1*, *DUSP1*, *EBF1, PDGFRB* expressing clusters) from the dataset. We re-clustered the nuclei and repeated this process if needed until no such clusters were found. We then used canonical marker genes (for example, *GAD1* for inhibitory neurons)^30,32,67,98^ to broadly annotate striatal nuclei in each species. Major cell types were defined as: SPNs, interneurons, astrocytes, MOLs, OPCs, and microglia.

After broad annotation, we extracted each broad category (for example, SPNs) from all species and integrated them using the default approach in Seurat v3 across all samples (SelectIntegrationFeatures, PrepSCTIntegration, FindIntegrationAnchors). During SPN filtering and annotation, we removed samples with less than 300 D1 SPN or D2 SPNs (for example, human sample Sample 242939, macaque samples SRR13808459, SRR13808461, SRR13808466, and SRR13808467 were removed). We annotated the SPN types and matrix and striosome clusters based on the canonical markers^75,98^. During interneuron filtering and annotation, we removed clusters that were not consistently present across all the groups within each species-tissue pair. We annotated the interneuron cell types based on the canonical markers^32,67^. For both SPN and interneuron integrations, only protein-coding genes that were orthologous across all the species were used. The orthologous genes were obtained using the NCBI Datasets tool (v 16.35.1).

We have calculated cell type proportions by dividing the total number of cells per cell type per individual to the total number of cells from the same individual, thus effectively accounting for the differences in cell recovery efficiency in different batches. We compared the means of these sample-level ratios across species/tissues using the compare_means function in R to perform t-tests. The nominal *p*-values were adjusted for multiple comparisons using the Benjamini–Hochberg false discovery rate (FDR) correction with FDR ≤ 0.05 threshold as well as scCODA^56^, where an FDR threshold of ≤0.1 was applied. Comparisons that achieved significance in both tests, considered statistically significant. The mean proportions, standard deviations, sample numbers, and standard errors of mean for each tissue-species pair are provided (Supplementary Data 2).

### Differentially expressed gene (DEG) and correlation analysis

When finding the conserved eSPN markers, we performed DEG analysis using FindMarkers from the Seurat package (Wilcoxon rank sum test) by comparing eSPN and other cells. To remove the neuron-specific DEGs and only focus on the eSPN-specific ones, we additionally performed DEG analysis by running FindMarkers using eSPN and other neurons (D1 SPNs, D2 SPNs, and interneurons). We then found the intersection of these lists of DEGs to find the eSPN-specific DEGs for that species. We repeated this process for all species. We then got the intersection of the eSPN-DEGs for all the species and filtered these DEGs by keeping the genes with scaled average expression greater than 1 for eSPN and plotted in a dot plot (Fig. 3F).

Hierarchical clustering was performed with the BuildClusterTree function of Seurat v3 with the default parameters: The centroids (mean values of the principal components) for each cluster were calculated to reduce the dataset to one representative vector per cluster. The pairwise Euclidean distances between the clusters were calculated, and using the Ward D2 method to minimize the variance within each cluster, the distance matrix was created. This distance matrix was visualized using the PlotClusterTree function.

Similarly to eSPN DEGs, we used FindMarkers from Seurat (Wilcoxon rank sum test) to determine the *LMO3* and *FOXP2 TSHZ2* interneuron specific DEGs within bat putamen compared to all other interneurons and showed the statistically upregulated (log_2_ fold change > 0.6 adjusted *p* value < 0.05) and downregulated (log_2_ fold change < −0.6 adjusted *p* value < 0.05) genes in the volcano plots (Fig. 4F).

Pearson correlation analysis with default parameters was performed to assess the similarity between gene expression profiles of D1–D2 hybrid cells from the macaque dataset^48^ and our SPN dataset, as well as interneuron cell types within bat Pu. The resulting correlation values were visualized with a heatmap (Figs. S3G and 4G).

### Bat interneuron subtype analyses

We performed weighted gene correlation network analysis (WGCNA) within bat putamen interneurons to identify co-expressed genes within each interneuron subtype^64^. Prior to running WGCNA, to increase statistical power for subtypes with fewer than 100 cells per sample, we merged certain cell populations: *PDGFD PTHLH PVALB*+, *PDGFD PTHLH PVALB-*, and *PDGFD* were combined into a single *PDGFD* group; *CCK VIP-* and *CCK VIP+* were combined as *CCK*; and *TAC3* and *TH* cells were merged as *TAC3-TH*. WGCNA was then performed using the log_2_ normalized counts; the same genes were later used in FindAllMarkers to detect the interneuron subtype-specific genes. Signed networks were used to determine the module genes. A soft-threshold power was automatically calculated to achieve approximate scale-free topology (*R*^2^ > 0.85). Networks were constructed with the blockwiseModules function with biweight midcorrelation (bicor). with the following modifications: maxBlockSize = 15,000, deepSplit = 0.5, minCoreKME = 0.4, minKMEtoStay = 0.5. We performed Fisher’s exact test to assess the enrichment between the gene modules and the markers found by FindAllMarkers (logfc.threshold  = 0.5, *p*_val_adj < 0.05, and avg_log2FC > 0) for each subtype. The –log₁₀ of FDR-adjusted *p*-values and the corresponding odds ratios were reported for each module-subtype marker enrichment. We used the top 200 connections for visualization^99^. We performed gene ontology (GO) enrichment analysis using enrichGO^100^ with the following parameters: universe = all genes tested in DEG analysis for that cell type, OrgDb = org.Hs.eg.db, keyType  = "SYMBOL,” ont = “MF,” pAdjustMethod = "BH,” *p*valueCutoff = 0.05, qvalueCutoff = 0.2).

### Human-specific differentially expressed gene (HS DEG) analysis

We calculated the human-specifically expressed genes as follows: we first generated a pseudobulk of gene expression counts of all the samples, performed DESeq2 in a pairwise fashion (chimp vs. human, etc.) using the following covariates: sex, log10 of the sum of total RNA, humanized age, and species. We performed an intersection of all the significant genes among each comparison in an upset plot. To find the HS DEGs, we intersected the significant (FDR < 0.05 and log2FC > 0.5) DEGs between human and chimpanzee with the significant DEGs between human and macaque and between human and marmoset and the non-significant DEGs between chimpanzee and macaque (FDR > 0.1).

We calculated the proportions of HS DEGs to all genes tested in the DE analysis for each cell type. Then we tested the difference in these proportions using the chi-square test. FDR < 0.05 was considered significant. We removed interneurons during these calculations as they are highly heterogenous, and we do not have enough power to test each interneuron subtype separately.

WGCNA, marker gene enrichment analysis between modules and cell type-specific HS DEGs, as well as GO-term enrichment analysis were performed, following the same approach and cutoffs as the bat interneuron WGCNA and GO-term analyses.

### Single-molecule fluorescence in situ hybridization (smFISH)

smFISH was performed using RNAScope Multiplex v2 Fluorescent assay. All steps were performed according to the manufacturer’s instructions (fresh frozen tissues for human and fixed frozen tissues for bats and mice), but with the addition of Sudan Black B. Sudan Black B (0.05%) added to the tissues after application of DAPI (Advanced Cell Diagnostics (ACD) #320858) to quench autofluorescence. We used the following: bat *MOG* probe (ACD #1279921-C2) with opal dye 570 (Akoya Biosciences #NC1601878), human *MOG* (ACD #543181-C2) with opal dye 570 (Akoya Biosciences #NC1601878), bat *RBFOX3* probe (ACD #1279931-C3) with opal dye 690 (Akoya Biosciences #NC1605064), human *RBFOX3* probe (ACD #415591-C3) with opal dye 690 (Akoya Biosciences #NC1605064), human *DRD1* (ACD #524991-C2) with opal dye 570 (Akoya Biosciences #NC1601878), human *DRD2* (ACD #553991-C3) with opal dye 520 (Akoya Biosciences #NC1601877), human *CASZ1* (ACD #882211) with opal dye 690 (Akoya Biosciences #NC1605064), mouse *Drd1* (ACD #461901) with opal dye 570 (Akoya Biosciences #NC1601878), mouse *Drd2* (ACD #406501-C2) with opal dye 520 (Akoya Biosciences #NC1601877), and mouse *Casz1* (ACD #502461-C3) with opal dye 690 (Akoya Biosciences #NC1605064).

Imaging *RBFOX3* and *MOG* was performed at ×20 magnification (zoom 2.5), and imaging *DRD1*, *DRD2*, and *CASZ1* was performed at ×20 magnification (zoom 1 and zoom 2.5) using a Zeiss LSM 880 in the UT Southwestern Neuroscience Microscopy Facility.

Cell numbers (*MOG*+, *RBFOX3*+, etc.) were quantified manually in a double-blind manner using Fiji. For example, MOG was defined as double positive cells for *MOG* and DAPI, RBFOX3 was defined as double positive cells for *RBFOX3* and DAPI. To compare neuronal and oligodendrocyte composition in the caudate between humans (*n* = 4) and bats (*n* = 4), between the caudate (*n* = 4) and putamen (*n* = 4) in bats, and to assess the eSPN/SPN ratio between humans (*n* = 2) and mice (*n* = 3), we obtained at least two measurements per individual (Supplementary Data 1). We quantified CASZ1+/DRD1+ cells (eSPNs) and DRD1+ or DRD2+ cells (SPNs) in the caudate (human) and caudate-putamen (mouse). Data were analyzed using a linear mixed model with species as the fixed factor and individual as the random factor (lme4 package in R, with REML = FALSE). Reported *p*-values are the main effect of species/tissue from a linear mixed model (two-sided). Statistical significance was set at FDR *p* < 0.05.

### Reporting summary

Further information on research design is available in the Nature Portfolio Reporting Summary linked to this article.

## Supplementary information

Supplementary Information

Description of Additional Supplementary Files

Supplementary Data 1

Supplementary Data 2

Supplementary Data 3

Supplementary Data 4

Supplementary Data 5

Supplementary Data 6

Supplementary Data 7

Supplementary Data 8

Supplementary Data 9

Supplementary Data 10

Reporting Summary

Transparent Peer Review File

## Source data

Source Data

## Data Availability

The human, chimpanzee, and pale spear-nosed bat dorsal striatum snRNA-seq data generated in this study have been deposited in the GEO database under accession code GSE293075. The processed data are available at UCSC cell browser [https://mammal-striatum-evo.cells.ucsc.edu]. The following published dorsal striatum snRNA-seq data used in this study were downloaded from the GEO database: rhesus macaque dataset with accession number GSE167920, marmoset datasets with accession numbers GSE151761 and GSE165578, mouse and ferret datasets with accession number GSE151761. Source data are provided with this paper.

## Code availability

All the scripts used in the study were deposited to https://github.com/konopkalab/Comparative_striatum and Zenodo^101^.
