# Supplementary material for: Comparative analysis of the cellular landscape in mammalian striatum
Source: Nat Commun. 2026 May 25;17:6793. doi: 10.1038/s41467-026-73305-8 (PMC13385833; doi:10.1038/s41467-026-73305-8)
Supplement: Supplementary file 1 — Supplementary Information [file 41467_2026_73305_MOESM1_ESM.pdf]

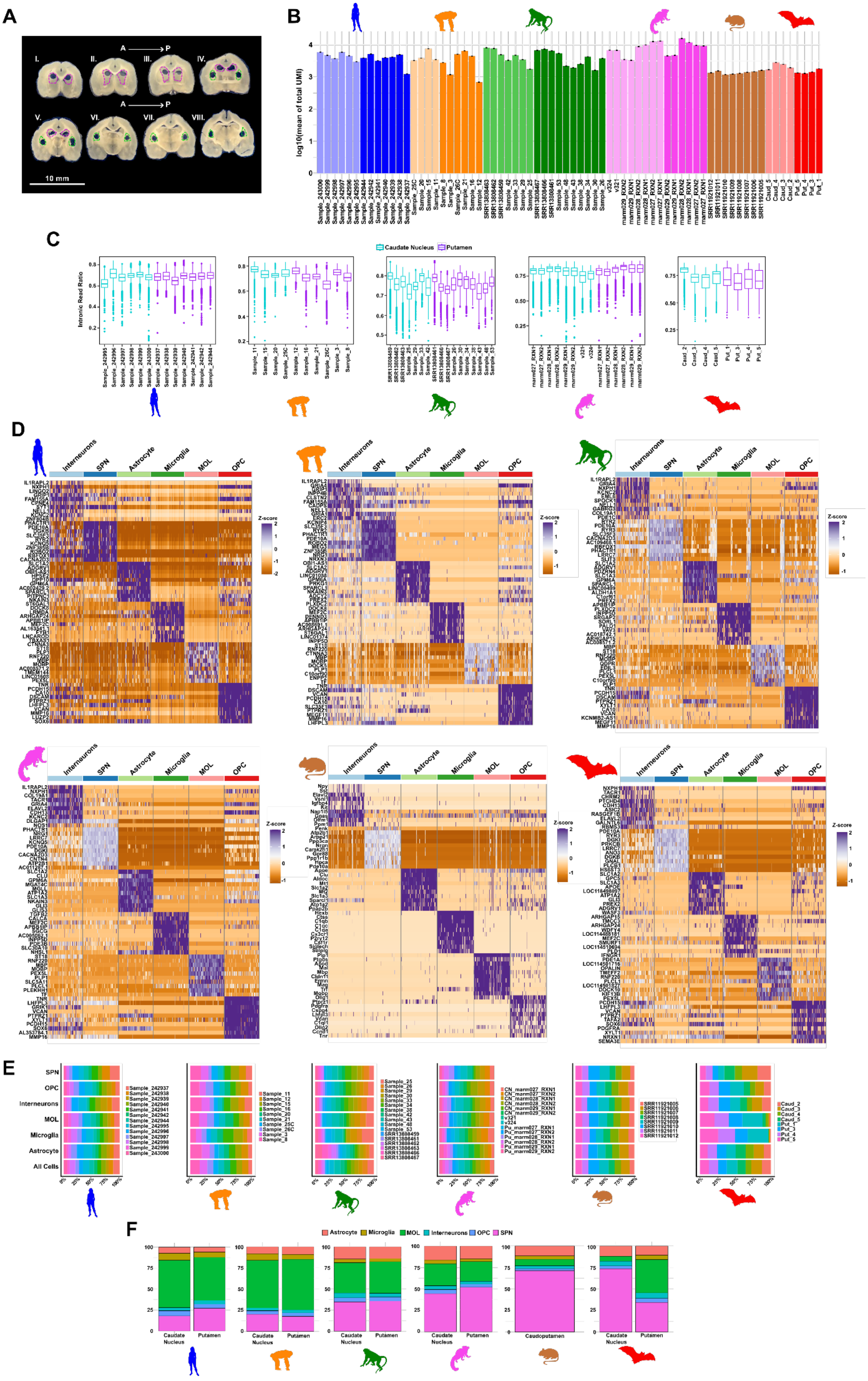
A
I.
II.
III.
IV.
V.
VI.
VII.
VIII.
A → P
10 mm
B
log10(mean of total UMI)
C
Caudate Nucleus
Putamen
Intronic Read Ratio
D
Interneurons
SPN
Astrocyte
Microglia
MOL
OPC
Z-score
E
SPN
OPC
Interneurons
MOL
Microglia
Astrocyte
All Cells
F
Astrocyte
Microglia
MOL
Interneurons
OPC
SPN
Caudate Nucleus
Putamen
Caudoputamen

Fig.S1. Quality control metrics for major cell type annotations across species. A. Representative photos of bat brain tissue dissection. Punches were made from the coronal sections of bat CN and Pu. Drawings in magenta indicate the punches made through the CN (I-IV) and drawings in green indicate the punches made through the Pu (IV-VIII). The scale bar marks 10 mm. B. Bar plots illustrate the mean total number of unique molecular identifiers (UMIs) per cell across samples, displayed on a logarithmic scale (base 10), segmented by species and tissue. The light colors represent the CN and the dark colors represent either Pu or C-Pu. C. Box plots depicting the intronic read ratios for samples, across all species. Boxplots show the median (center line), interquartile range (box), and whiskers indicating the minimum and maximum values within 1.5× the interquartile range. D. Heatmap depicting the scaled (z-score) expression levels of the top 10 most highly differentially expressed genes shown for the randomly sampled 200 nuclei within each major cell type annotation in each species. E. Cell type composition of each sample across species. F. Bar plots displaying the proportional composition of major cell type annotations within each tissue type (CN, Pu, caudoputamen) and species. CN: caudate nucleus, Pu: putamen. A: anterior. P: posterior. Source data are provided as a Source Data file. Animal silhouettes from PhyloPic (chimpanzee: Caspar K.R.; Phyllostomus discolor: Sibaja R.D.; mouse: Miranda-Rottmann S.), used under CC-BY 3.0 (https://creativecommons.org/licenses/by/3.0/); images recolored.

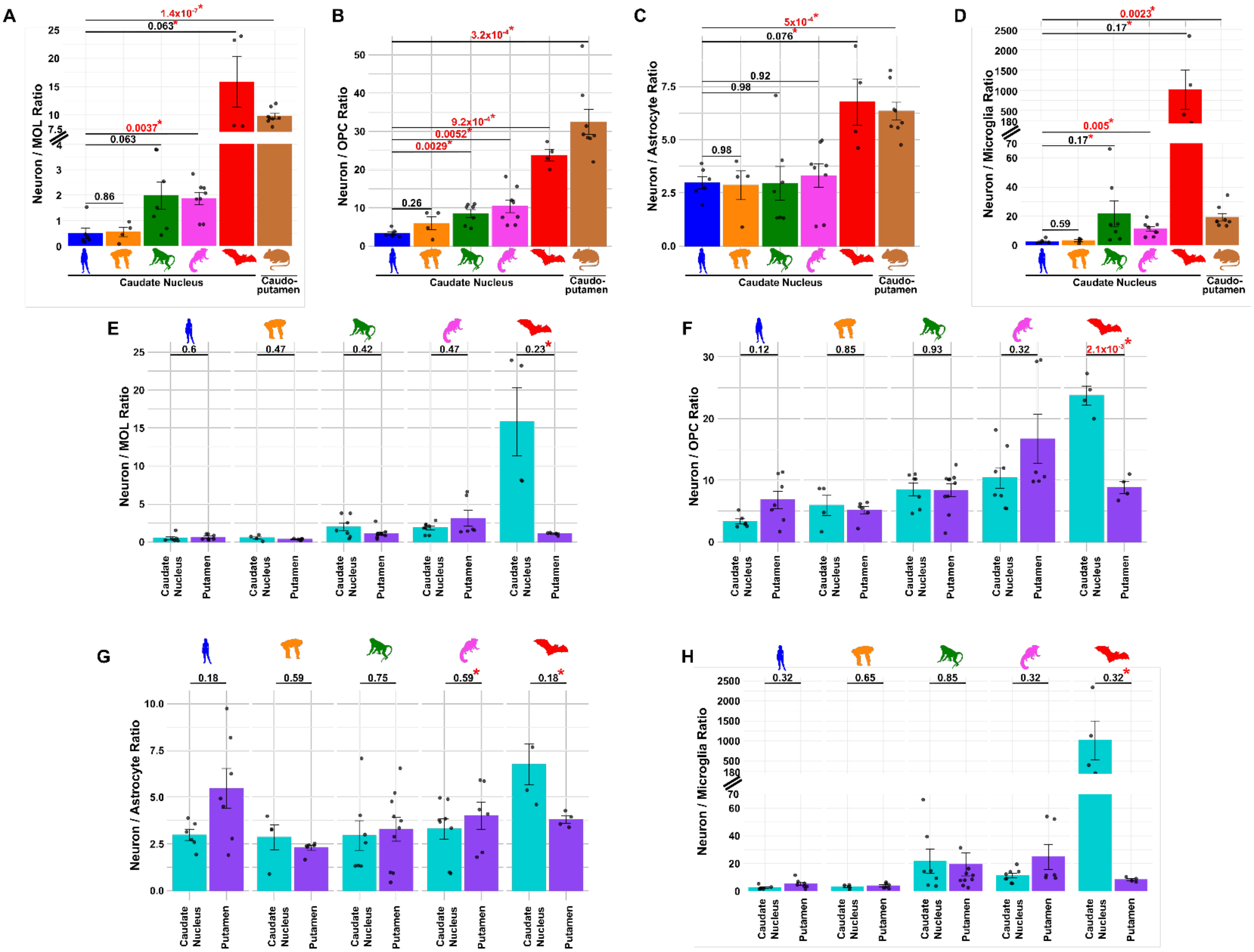

Fig.S2. Ratios of neuron to subtypes of glia. A-D. Bar plots illustrating the ratios of neuron counts to counts of various glial subtypes: A. Mature oligodendrocytes (MOL), B. Oligodendrocyte progenitor cells (OPC), C. Astrocytes, and D. Microglia. These ratios are depicted for the caudate nucleus (CN) across all species, and the caudoputamen in mouse. E-H. Bar plots displaying the ratios of neuron counts to counts of glial subtypes in the following categories: E. Mature oligodendrocytes (MOL), F. Oligodendrocyte progenitor cells (OPC), G. Astrocytes, and H. Microglia. These ratios are shown for both the CN and putamen (Pu) across all species. Two tailed t-test was used and nominal p values were multi-test corrected using Benjamini-Hochberg method to assess statistical significance between the groups (FDR < 0.05 is considered significant and written in red). Additionally, red asterisk indicates that the comparison is statistically significant in scCODA analysis. Error bars indicate mean ± standard error of the mean. Source data are provided as a Source Data file. Animal silhouettes from PhyloPic (chimpanzee: Caspar K.R.; Phyllostomus discolor: Sibaja R.D.; mouse: Miranda-Rottmann S.), used under CC-BY 3.0 (https://creativecommons.org/licenses/by/3.0/); images recolored.

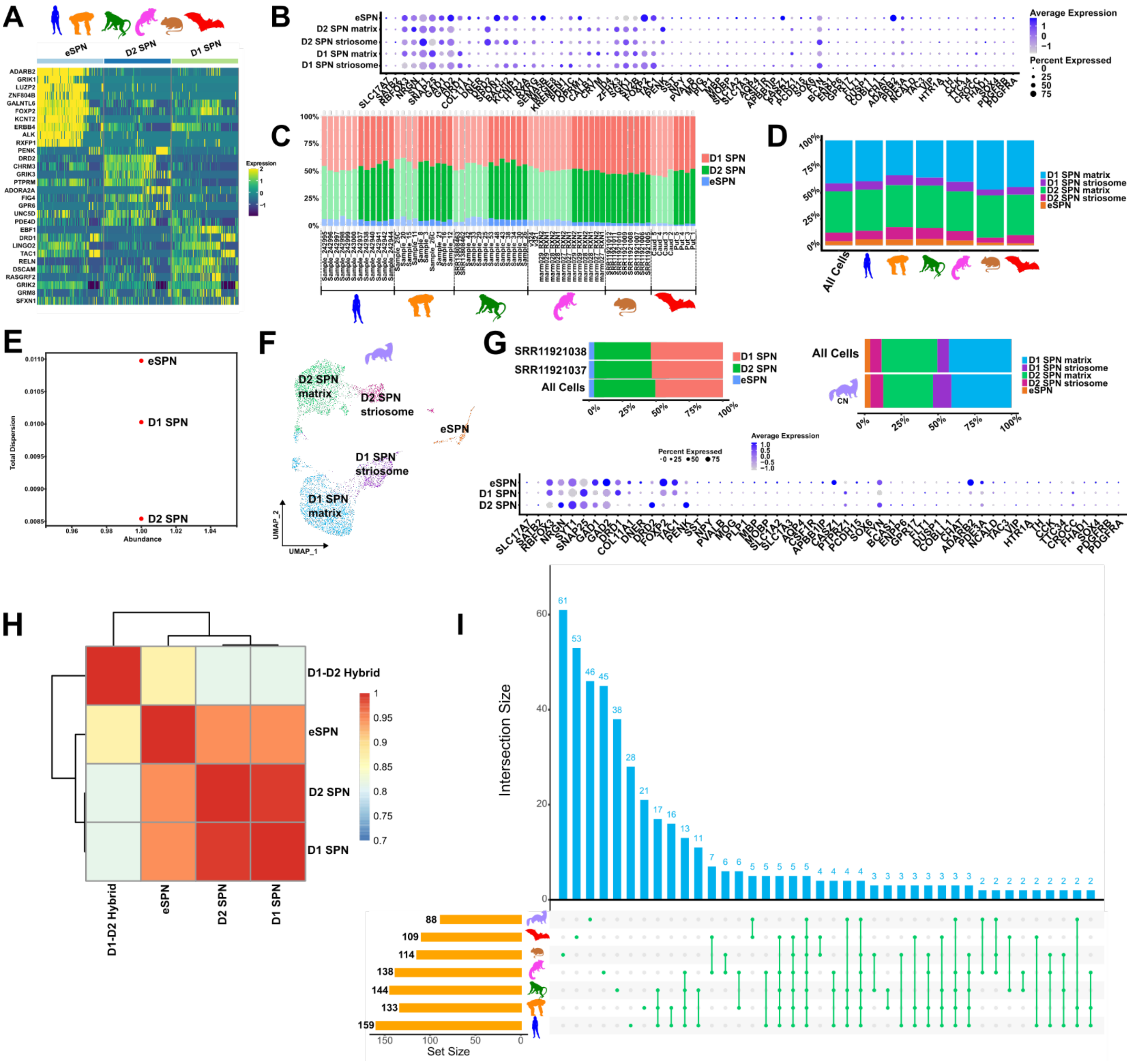

Fig.S3. Spiny projection neuron (SPN) characteristics. A. Heatmap showing the transcriptomic profile of the top 10 genes in each SPN subtype: D1 SPN, D2 SPN, and eSPN across human, chimpanzee, rhesus macaque, marmoset, mouse, and bat. B. Dot plot of marker genes used to determine the SPN subtypes: D1 SPN striosome, D1 SPN matrix, D2 SPN striosome, D2 SPN matrix, and eSPN. C. Stacked bar plot depicting the percentages of each SPN subtype (D1 SPN, D2 SPN, and eSPN) across samples. The samples were grouped together according to the species they belong to. The light colors represent the CN and the dark colors represent either Pu or C-Pu. D. Cellular composition of SPN subtypes: D1 SPN striosome, D1 SPN matrix, D2 SPN striosome, D2 SPN matrix, and eSPN across human, chimpanzee, rhesus macaque, marmoset, mouse, and bat. E. Dispersion and cellular abundance plots for SPNs. The SPN subtypes indicated in red were found to be higher than 0.9 in abundance. F. UMAP of SPNs of ferret CN (n=2). G. The stacked bar plot of SPN subtypes: D1 SPN, D2 SPN, and eSPN across ferret samples and the cellular composition of SPN subtypes: D1 SPN striosome, D1 SPN matrix, D2 SPN striosome, D2 SPN matrix, and eSPN in ferret dataset. Dot plot of the marker genes in the ferret dataset. H. Heatmap showing the Pearson correlation matrix between the normalized gene counts of the D1-D2 hybrid cells in the published macaque dataset (He, Kleyman et al. 2021) and our SPN dataset. The dendrogram depicts the hierarchical clustering of these cells. I. Upset plot of differentially expressed genes (DEGs) in eSPNs compared to other cell types across species: human, chimpanzee, rhesus macaque, marmoset, mouse, bat, and ferret. Each blue bar indicates the number of common DEGs for the indicated combination of species in green. Yellow bars indicate the total number of eSPN DEGs found in each species. Source data are provided as a Source Data file.

Animal silhouettes from PhyloPic (chimpanzee: Caspar K.R.; Phyllostomus discolor: Sibaja R.D.; mouse: Miranda-Rottmann S.), used under CC-BY 3.0 (https://creativecommons.org/licenses/by/3.0/); images recolored.

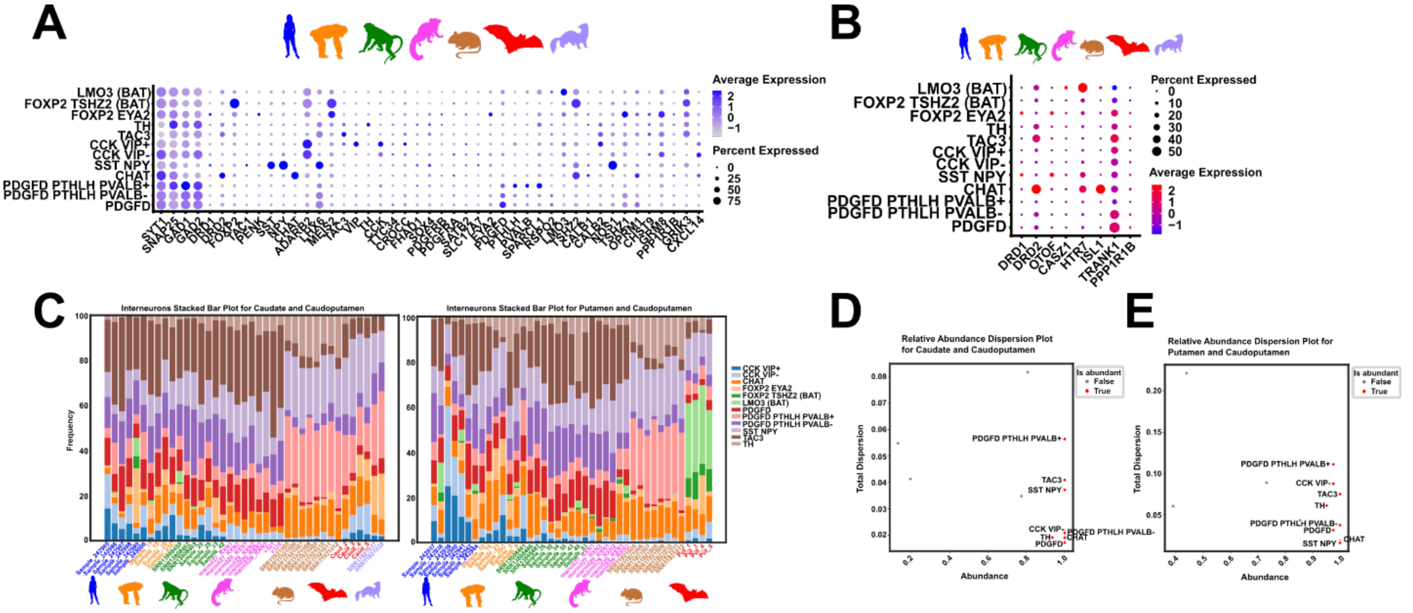

Fig.S4. Striatal interneurons across all species. A. Dot plot showing the expression of marker genes across striatal interneuron cell types of human, chimpanzee, rhesus macaque, marmoset, mouse, bat, and ferret. B. Dot plot showing the expressions of SPN marker genes across striatal interneuron cell types of human, chimpanzee, rhesus macaque, marmoset, mouse, bat, and ferret. C. Sample level distribution of striatal interneuron cell types in CN and C-Pu and in Pu and C-Pu. D-E. Dispersion and cellular abundance plots for D. CN and E. Pu. The striatal interneuron cell types indicated in red were found to be higher than 0.9 in abundance. CN: Caudate Nucleus, Pu: Putamen, C-Pu: Caudoputamen. Animal silhouettes from PhyloPic (chimpanzee: Caspar K.R.; Phyllostomus discolor: Sibaja R.D.; mouse: Miranda-Rottmann S.), used under CC-BY 3.0 (https://creativecommons.org/licenses/by/3.0/); images recolored.
