# Supplementary material for: Comparative analysis of the cellular landscape in mammalian striatum
Source: Nat Commun. 2026 May 25;17:6793. doi: 10.1038/s41467-026-73305-8 (PMC13385833; doi:10.1038/s41467-026-73305-8)
Supplement: Supplementary file 2 — Description of Additional Supplementary Files [file 41467_2026_73305_MOESM2_ESM.pdf]

File name: Supplementary Data 1

Description: Sample metadata for each species and smFISH experiments.

File name: Supplementary Data 2

Description: Summary of neuronal proportions across species and tissues, including scCODA results for neuron-to-glia and neuron-to-glia subtype comparisons across species and tissues.

File name: Supplementary Data 3

Description: scCODA results for interneuron proportions across species in the putamen.

File name: Supplementary Data 4

Description: Interneuron proportions across samples, species, and tissues, including scCODA results comparing primates and non-primates across tissues.

File name: Supplementary Data 5

Description: Differential gene expression analysis results of *LMO3 (BAT)* interneurons compared to other interneurons within the bat putamen. Significantly upregulated and downregulated genes were identified using a two-sided Wilcoxon rank-sum test with Bonferroni adjustment ($P < 0.05$; log2FC > 0.6 for upregulated genes and log2FC < −0.6 for downregulated genes).

File name: Supplementary Data 6

Description: Differential gene expression analysis results of *FOXP2 TSHZ2 (BAT)* interneurons compared to other interneurons within the bat putamen. Significantly upregulated and downregulated genes were identified using a two-sided Wilcoxon rank-sum test with Bonferroni adjustment ($P < 0.05$; log2FC > 0.6 for upregulated genes and log2FC < −0.6 for downregulated genes).

File name: Supplementary Data 7

Description: Gene ontology enrichment analysis of WGCNA results for bat cell types was performed using a one-sided hypergeometric test. Terms with FDR < 0.01 were considered significant.

File name: Supplementary Data 8

Description: scCODA results for interneuron proportions across species in the caudate nucleus, with human as the reference.

File name: Supplementary Data 9

Description: scCODA results for interneuron proportions across species in the putamen, with human as the reference.

File name: Supplementary Data 10

Description: Gene ontology enrichment analysis of WGCNA results for human cell types was performed using a one-sided hypergeometric test. Terms with FDR < 0.01 were considered significant.
