# Supplementary material for: Comparative analysis of the cellular landscape in mammalian striatum
Source: Nat Commun. 2026 May 25;17:6793. doi: 10.1038/s41467-026-73305-8 (PMC13385833; doi:10.1038/s41467-026-73305-8)
Supplement: Supplementary file 13 — Reporting Summary [file 41467_2026_73305_MOESM13_ESM.pdf]

nature portfolio

Corresponding author(s): Konopka, Genevieve & Vernes, Sonja

Last updated by author(s): Apr 16, 2026

Reporting Summary

Nature Portfolio wishes to improve the reproducibility of the work that we publish. This form provides structure for consistency and transparency in reporting. For further information on Nature Portfolio policies, see our Editorial Policies and the Editorial Policy Checklist.

Statistics

For all statistical analyses, confirm that the following items are present in the figure legend, table legend, main text, or Methods section.

n/a	Confirmed	
☐	☒	The exact sample size (*n*) for each experimental group/condition, given as a discrete number and unit of measurement
☐	☒	A statement on whether measurements were taken from distinct samples or whether the same sample was measured repeatedly
☐	☒	The statistical test(s) used AND whether they are one- or two-sided<br>*Only common tests should be described solely by name; describe more complex techniques in the Methods section.*
☐	☒	A description of all covariates tested
☐	☒	A description of any assumptions or corrections, such as tests of normality and adjustment for multiple comparisons
☐	☒	A full description of the statistical parameters including central tendency (e.g. means) or other basic estimates (e.g. regression coefficient) AND variation (e.g. standard deviation) or associated estimates of uncertainty (e.g. confidence intervals)
☐	☒	For null hypothesis testing, the test statistic (e.g. *F*, *t*, *r*) with confidence intervals, effect sizes, degrees of freedom and *P* value noted<br>*Give P values as exact values whenever suitable.*
☐	☒	For Bayesian analysis, information on the choice of priors and Markov chain Monte Carlo settings
☐	☒	For hierarchical and complex designs, identification of the appropriate level for tests and full reporting of outcomes
☐	☒	Estimates of effect sizes (e.g. Cohen's *d*, Pearson's *r*), indicating how they were calculated

Our web collection on statistics for biologists contains articles on many of the points above.

Software and code

Policy information about availability of computer code

Data collection	R (v 4.2.3) was used to process and analyze single-nuclear RNA Sequencing data
Data analysis	Datasets were generated as detailed in the Methods. Alignment to each species own reference genome was done using cellranger software (namely cellranger mkfastq and cellranger count) and Cellbender was used to obtain the final gene-cell as detailed in the analysis.<br>Gene-cell matrix across all species were post-processed for custom analyses as detailed in the Methods. For each analyses, we explicitly report the tools and our approach. We also provide the full script for full transparency. Here is the list and versions of standalone tools we have used:<br><br>Cellranger v6.0.0,<br>CellBender v0.1.0,<br>NCBI Datasets tool v16.35.1<br>scCODA (https://github.com/theislab/scCODA)<br><br>Major R libraries used:<br>Seurat (v4.4.0), GeneOverlap(v1.34.0), harmony (v1.0.1), ggplot2(v3.4.4)<br><br>Custom R and python codes used for this analysis are deposited and available at:<br>https://github.com/konopkalab/Comparative_striatum

For manuscripts utilizing custom algorithms or software that are central to the research but not yet described in published literature, software must be made available to editors and reviewers. We strongly encourage code deposition in a community repository (e.g. GitHub). See the Nature Portfolio guidelines for submitting code & software for further information.

Data

Policy information about availability of data

All manuscripts must include a data availability statement. This statement should provide the following information, where applicable:

- Accession codes, unique identifiers, or web links for publicly available datasets
- A description of any restrictions on data availability
- For clinical datasets or third party data, please ensure that the statement adheres to our policy

Raw and processed human, chimpanzee, and pale spear-nosed bat dorsal striatum snRNA-seq datasets were deposited to GEO, accession number GSE293075. The following published dorsal striatum snRNA-seq datasets were downloaded from GEO and used in the analysis: Rhesus macaque dataset with accession number GSE167920, marmoset datasets with accession numbers GSE151761 and GSE165578, mouse and ferret datasets with accession number GSE151761.

Research involving human participants, their data, or biological material

Policy information about studies with human participants or human data. See also policy information about sex, gender (identity/presentation), and sexual orientation and race, ethnicity and racism.

Reporting on sex and gender	No sex-based analyses were performed, as this study focuses on species-level comparisons of gene expression and cellular composition rather than sex-specific effects.
Reporting on race, ethnicity, or other socially relevant groupings	No ethnicity-based analyses were performed.
Population characteristics	N/A
Recruitment	N/A
Ethics oversight	UT Southwestern Medical Center Institutional Review Board has determined that as this research was conducted using postmortem specimens, the project does not meet the definition of human subjects research and does not require IRB approval and oversight.

Note that full information on the approval of the study protocol must also be provided in the manuscript.

Field-specific reporting

Please select the one below that is the best fit for your research. If you are not sure, read the appropriate sections before making your selection.

[x] Life sciences [ ] Behavioural & social sciences [ ] Ecological, evolutionary & environmental sciences

For a reference copy of the document with all sections, see nature.com/documents/nr-reporting-summary-flat.pdf

Life sciences study design

All studies must disclose on these points even when the disclosure is negative.

Sample size	No statistical methods were used to predetermine sample size for the datasets we generated. However, our sample sizes for single nuclear RNA-seq is similar to other published studies.
Data exclusions	Data that represented ambient RNA and/or doublets were excluded. This exclusion criteria was pre-established.
Replication	We replicated key findings related to neuron-to-glia ratios and eSPN-to-SPN ratios in other published dorsal striatum datasets (rhesus macaque, marmoset, mouse, ferret, etc) as well as by single molecule fluorescence in situ hybridization (smFISH) .
Randomization	Samples were not randomized. All known technical and biological covariates were included in the statistical models, and therefore randomization is not relevant.
Blinding	Data collection and analysis were not performed blind to the conditions of the experiments. Data collection and analysis were carried out by different individuals. For collection, in order for samples to be assigned to the correct group (human, chimpanzee, rhesus macaque, and bat), knowledge of species could not be blind. For analysis, in order to align reads to the correct genome, we needed to know which samples belonged to each species. However counting of smFISH results were performed in a blinded fashion.

Reporting for specific materials, systems and methods

We require information from authors about some types of materials, experimental systems and methods used in many studies. Here, indicate whether each material, system or method listed is relevant to your study. If you are not sure if a list item applies to your research, read the appropriate section before selecting a response.

Materials & experimental systems

n/a	Involved in the study
☒	☐ Antibodies
☒	☐ Eukaryotic cell lines
☒	☐ Palaeontology and archaeology
☐	☒ Animals and other organisms
☒	☐ Clinical data
☒	☐ Dual use research of concern
☒	☐ Plants

Methods

n/a	Involved in the study
☒	☐ ChIP-seq
☒	☐ Flow cytometry
☒	☐ MRI-based neuroimaging

Animals and other research organisms

Policy information about studies involving animals; ARRIVE guidelines recommended for reporting animal research, and Sex and Gender in Research

Laboratory animals	All relevant details of samples are described and in the Source data file, Table S1.
Wild animals	This study did not involve wild animals.
Reporting on sex	No sex-based analyses were performed, as this study focuses on species-level comparisons of gene expression and cellular composition rather than sex-specific effects.
Field-collected samples	This study did not involve field-collected samples.
Ethics oversight	All use of Non-human primate material was approved by UT Southwestern Medical Center Institutional Biosafety Committee (NHMSR-2021-012). The pale spear-nosed bat animal care and tissue collection were complied with the principles of laboratory animal care and the regulations of the current version of the German Law on Animal Protection and the Animal Scientific Procedures Act (1986) under Home Office (UK) supervision. The number of animals used in terminal experiments was reported to the Munich veterinary office and the Home Office (UK).

Note that full information on the approval of the study protocol must also be provided in the manuscript.

Plants

Seed stocks	*Report on the source of all seed stocks or other plant material used. If applicable, state the seed stock centre and catalogue number. If plant specimens were collected from the field, describe the collection location, date and sampling procedures.*
Novel plant genotypes	*Describe the methods by which all novel plant genotypes were produced. This includes those generated by transgenic approaches, gene editing, chemical/radiation-based mutagenesis and hybridization. For transgenic lines, describe the transformation method, the number of independent lines analyzed and the generation upon which experiments were performed. For gene-edited lines, describe the editor used, the endogenous sequence targeted for editing, the targeting guide RNA sequence (if applicable) and how the editor was applied.*
Authentication	*Describe any authentication procedures for each seed stock used or novel genotype generated. Describe any experiments used to assess the effect of a mutation and, where applicable, how potential secondary effects (e.g. second site T-DNA insertions, mosiacism, off-target gene editing) were examined.*
