# Supplementary material for: Comparative analysis of the cellular landscape in mammalian striatum
Source: Nat Commun. 2026 May 25;17:6793. doi: 10.1038/s41467-026-73305-8 (PMC13385833; doi:10.1038/s41467-026-73305-8)
Supplement: Supplementary file 14 — Transparent Peer Review File [file 41467_2026_73305_MOESM14_ESM.pdf]

nature portfolio

Peer Review File

Comparative analysis of the cellular landscape in mammalian striatum

Corresponding Author: Dr Genevieve Konopka

This file contains all reviewer reports in order by version, followed by all author rebuttals in order by version.

Version 0:

Reviewer comments:

Reviewer #1

(Remarks to the Author)
In this work Buyukkahraman et al. have carried out a comparative analysis of snRNAseq datasets from the dorsal striatum of different species, including human, chimpanzee, macaque, and bat (datasets generated by the authors) as well as marmoset, mouse and ferret (from previous studies). They provide an overview of the relative abundance of cell types across species and between the two structures that comprise the dorsal striatum—the putamen (Pu) and caudate nucleus (CN)—and then focus specifically on neurons, particularly interneurons. Among the main findings they reported a significantly higher proportion in the CN of macaques and marmosets compared to humans and chimpanzees, a difference not observed in the Pu, a markedly lower neuron-to-glia ratio in the primate striatum relative to non-primate species, and two interneuron bat-specific classes. These findings represent a valuable contribution to the field of comparative neurobiology and transcriptomics, particularly given the novelty of the bat and chimpanzee datasets. However, beyond the presentation of these datasets, the study lacks major conceptual advances or conclusions for publication in Nature Communications. In addition, several key aspects of the writing, analysis (particularly the statistical methodology), and data presentation require clarification and further detail to fully support the authors' claims. Below, I present specific concerns that highlight the fundamental limitations of the study.

Main points.

1. Clarity in the scope of the study.
The study claims to be an evolutionary analysis, as stated in the title, but the work presented is essentially a brief comparative analysis of striatal snRNA-seq datasets across species. There are no evolution-driven analyses included. The title—and corresponding sections such as the Results and Discussion—should be revised accordingly to more accurately reflect the scope of the study.

2. Limited use of the transcriptomic data and absence of novel insights.
The analytical framework is heavily focused on compositional differences (i.e., relative abundances of cell types), which, although informative, remains largely descriptive. Additional layers of analysis—such as gene set enrichment, transcription factor activity profiling, or cell–cell interaction inference—would substantially enhance the functional interpretation of the findings and the study's overall contribution to the field.
Along similar lines, one of the main findings highlighted by the authors is the identification of two bat-specific interneuron subtypes, which is positioned as a central point in the Discussion. However, further analysis of these subtypes beyond the identification of marker genes would be valuable to better characterize their potential functional roles.
Overall, the study does not offer any major conceptual advances.

3. Limited sample size and inconsistent statistical analysis.
Although the integrated dataset spans several species, the number of biological replicates per species and region is limited for some of them (e.g., n = 4 for bat caudate nucleus). This constraint significantly reduces statistical power and makes many comparisons susceptible to noise. Another key limitation of the manuscript lies in the statistical framework used to support its central comparative claims. Several conclusions—such as differences in neuronal and glial proportions or eSPN/SPN ratios across species—are derived from pairwise two-tailed t-tests, without clear description of how values were aggregated (e.g., per individual vs. per cell), whether data were normalized, or whether corrections for multiple testing were applied. Notably,

ratio-based comparisons across species (e.g., eSPN/SPN and neuron/glia ratios) require normalization of input cell counts (e.g., controlling for total recovered cells per sample or tissue) to avoid biases introduced by differing cell recovery efficiencies across species or experimental batches. The absence or insufficient description of such normalization undermines the validity of these cross-species comparisons.
Additionally, the manuscript repeatedly applies uncorrected t-tests across multiple cell types and comparisons (e.g., Table S4; Figs. 2A, 3C–D), increasing the risk of false positives. Correction for multiple testing, such as using the Benjamini-Hochberg false discovery rate (FDR), should be implemented consistently to ensure statistical validity.

More robust compositional analysis methods—such as scCODA—are applied later in the manuscript, but inconsistently. For example, while scCODA is used for some analyses of SPN subtypes, it is not used to validate key comparisons such as neuronal/glial proportions or eSPN/SPN ratios, where its use would be most appropriate given the compositional nature of single-nucleus RNA-seq data. Importantly, in several cases, findings that are significant by t-test lose significance when tested using scCODA, highlighting the need for methodological consistency.

In summary, the inconsistent application of compositional methods, lack of clear normalization procedures in ratio-based analyses, and absence of multiple testing corrections collectively reduce the statistical rigor and interpretability of the results.

Minor Points.

1. Figure Clarity and Formatting.
-Standardize axis scales across comparable plots (e.g., Figs. 3C–D, 4E–F, 4H–I, S4D–E) to facilitate accurate visual comparisons.
-Maintain consistent species color schemes across figures (e.g., between Fig. 4A and 4C) to enhance interpretability.
-Improve layout alignment and spacing in multi-panel figures (e.g., Fig. 1C).
-Correct residual graphical artifacts (e.g., square-like borders, contour lines, "=" lines) in Supplementary Figures (e.g., S1C, S1D, S2A, S2D, S2H), likely due to vector-to-raster format conversions.
-Clarify whether expression heatmaps and figures showing negative values (e.g., Fig. 1B) reflect log, z-score, or other transformations; specify this in the figure legends and Methods. (Negative values are unexpected following standard normalization procedures commonly applied in sc/sn RNA-seq data processing)
-Supplementary Figure 1D lacks clarity, and the gene names are not readable.

2. Statistical Annotation and Reporting.
-Specify the summary statistic used in figures showing aggregated expression as it is the case of violin plots (e.g., mean vs. median in Fig. 1C).
-Report numerical values with appropriate statistical summaries (e.g., mean ± SEM or SD) when describing observed trends (e.g., Fig. 2A).
-For correlation analyses (e.g., Figs. 4G), report correlation coefficients (r) and associated p-values to support conclusions.

3. Validation and Statistical Claims.
-In smFISH validation (e.g., Figs. 2H–I), clearly describe the analysis in the Methods section, including the number of biological replicates and exact statistical tests used.
-Avoid claiming "significant" differences (e.g., in Fig. 4I, human vs. chimpanzee) when statistical significance is not achieved; instead, refer to such results as trends and provide actual p-values.
-Please avoid phrasing such as “the algorithm considered it not significant.” Instead, simply state whether the result is significant or not, along with the p-value and the test statistic.

4. Compositional Analyses.
Justify the choice of iSPNs as the reference population in scCODA analyses; ideally, add a supplementary figure showing their relative stability across samples and species.

5. Quality Control and Data Processing.
-Clarify whether the nuclear quality control (e.g., intronic read thresholds as shown in Fig. S1B) was the only QC step performed. If additional filters (e.g., mitochondrial gene content, total gene counts, or doublet detection) were used, these should be explicitly described.
-Detail the clustering methodology, including the algorithm (e.g., Louvain or Leiden), resolution used, and total number of clusters generated.
-Report how many clusters were excluded based on QC (e.g., low intronic ratios, high gene counts, or expression of non-neuronal markers like FLT1, DUSP1, EBF1, PDGFRB).
-Specify the normalization procedure applied (e.g., log-normalization, SCTransform); if no normalization was performed, this should be justified.

(Remarks on code availability)

I have reviewed the code, which includes a README file and the scripts used for the analysis; however, I have not executed it.

Reviewer #2

(Remarks to the Author)
In the present study Buyukkahraman et al. study the evolution of neuron types in the dorsal striatum. They used data from four primates - human, chimpanzee, macaque, and marmoset, and three nonprimate species - mice, bats, and ferrets. They found that primates had a lower neuron to glia ratio than non-primates, that primate DS contained a larger fraction of eSPNs, and they revealed some new interneuron types in the bat. The data appear to be analyzed properly, I liked the use of the liftover tool across species. That primate DS contains a larger fraction of eSPNs and the novel bat interneuron populations could constitute significant contributions. Still I have a couple of questions and concerns.

Major Concerns
The authors need to provide more details about the dissections. The drawing in figure 1 appears to indicate that human samples were taken from the rostral striatum, the He, Kleyman 2021 data they used is also rostral striatum, but the bat sampling shown in S1 takes cells broadly along the rostral-caudal extent. Can the 'novel' cell types in the bat be explained by the fact that they sampled more broadly? Moreover, these are the only species I could find any information for. The striatum is not a homogenous lump. More info needed.

A point somewhat related to my first concern is that the authors appear to give no consideration of functional differences between the species that could account for cell type differences. Primates have a huge caudate body because they have large associative and oculomotor cortical territories that are absent in other species. Bats, I imagine, have unique striatal regions dedicated to hearing. This point is similar to point one in that we need to ensure we are comparing apples to apples. However, it is slightly more subtle because, in an ideal world, we would only compare functionally homologous regions of the striatum. If that is not possible, then the authors should be more cautious with their claims.

The authors refer to dSPNs and iSPNs, but they don't show that the SPNs are actually part of the direct (d) and indirect (i) pathways. These authors are not the only ones to do this, but it still isn't accurate. Of course, most DRD1-expressing SPNs are part of the direct pathway, but there are some DRD1 neurons that project to GPe. I suggest that without data on the actual projection targets, we should annotate cell types with gene markers rather than assumptions.

(Remarks on code availability)

Reviewer #3

(Remarks to the Author)
I co-reviewed this manuscript with one of the reviewers who provided the listed reports. This is part of the Nature Communications initiative to facilitate training in peer review and to provide appropriate recognition for Early Career Researchers who co-review manuscripts.

(Remarks on code availability)

Version 1:

Reviewer comments:

Reviewer #1

(Remarks to the Author)
Thank you to the authors for the careful and detailed revision. The manuscript has improved, particularly in the methodological description, statistical clarification, and additional downstream analyses. The datasets generated—especially the new insights in bat —represent a valuable contribution that will be of interest to the community.
That said, after evaluating the revised manuscript and responses, I do not consider the work reaches the level of conceptual advance expected for publication in Nature Communications.
The authors have made a genuine effort to expand the analytical depth, including additional differential expression and WGCNA analyses for both the human lineage and the bat-specific interneurons. However, in my assessment, these additions remain largely descriptive and do not substantially elevate the central biological insight of the study.
A major remaining concern is the disconnect between the Results and the Discussion. While the Results now build a coherent narrative—from broad cellular proportions to human-specific transcriptomic changes (Fig. 5)—the Discussion does not meaningfully engage with these final molecular findings. In particular, the reported enrichment of human-specific DEGs in SPNs is not interpreted in a way that clarifies its functional or evolutionary significance. Likewise, although several highlighted genes (e.g., Tpbg, Hrh3, UNC80) are linked to neurological conditions, the manuscript does not adequately discuss what it might biologically imply that these genes show human-specific upregulation. As written, the conceptual implications of these analyses remain unclear.

Regarding the bat findings, the identification of putamen-specific interneuron subtypes is interesting and potentially important. The additional module analysis provides some characterization, but the functional interpretation remains limited, and the authors appropriately note the current knowledge gaps. Consequently the study remains short of delivering a strong mechanistic.
The authors have also improved the statistical framework and clarified normalization and multiple-testing procedures. These revisions address several of the prior technical concerns. However, even with these improvements, the overall advance of the manuscript remains primarily descriptive rather than conceptual.
In summary, I believe the datasets presented here will be a useful resource but in its current form the manuscript does not yet provide the level of conceptual insight and integrative interpretation expected for Nature Communications.
I hope these comments are helpful to the authors in further developing their work.

(Remarks on code availability)
The code was reviewed but not executed

Reviewer #2

(Remarks to the Author)
The authors have addressed my concerns.

(Remarks on code availability)

Reviewer #3

(Remarks to the Author)
I co-reviewed this manuscript with one of the reviewers who provided the listed reports. This is part of the Nature Communications initiative to facilitate training in peer review and to provide appropriate recognition for Early Career Researchers who co-review manuscripts.

(Remarks on code availability)

Open Access This Peer Review File is licensed under a Creative Commons Attribution 4.0 International License, which permits use, sharing, adaptation, distribution and reproduction in any medium or format, as long as you give appropriate credit to the original author(s) and the source, provide a link to the Creative Commons license, and indicate if changes were made.
In cases where reviewers are anonymous, credit should be given to 'Anonymous Referee' and the source.
The images or other third party material in this Peer Review File are included in the article's Creative Commons license, unless indicated otherwise in a credit line to the material. If material is not included in the article's Creative Commons license and your intended use is not permitted by statutory regulation or exceeds the permitted use, you will need to obtain permission directly from the copyright holder.

To view a copy of this license, visit https://creativecommons.org/licenses/by/4.0/

We thank the reviewers for their overall positive and helpful comments about our study. We appreciate the thoughtful suggestions and critiques. We have addressed each concern, resulting in what we believe is a significantly improved manuscript. All changes to the text are highlighted in yellow in the revised manuscript. Please see our point-by-point responses to each comment below.

REVIEWER COMMENTS

Reviewer #1 (Remarks to the Author):

In this work Buyukkahraman et al. have carried out a comparative analysis of snRNAseq datasets from the dorsal striatum of different species, including human, chimpanzee, macaque, and bat (datasets generated by the authors) as well as marmoset, mouse and ferret (from previous studies). They provide an overview of the relative abundance of cell types across species and between the two structures that comprise the dorsal striatum—the putamen (Pu) and caudate nucleus (CN)—and then focus specifically on neurons, particularly interneurons. Among the main findings they reported a significantly higher proportion in the CN of macaques and marmosets compared to humans and chimpanzees, a difference not observed in the Pu, a markedly lower neuron-to-glia ratio in the primate striatum relative to non-primate species, and two interneuron bat-specific classes. These findings represent a valuable contribution to the field of comparative neurobiology and transcriptomics, particularly given the novelty of the bat and chimpanzee datasets. However, beyond the presentation of these datasets, the study lacks major conceptual advances or conclusions for publication in Nature Communications. In addition, several key aspects of the writing, analysis (particularly the statistical methodology), and data presentation require clarification and further detail to fully support the authors' claims. Below, I present specific concerns that highlight the fundamental limitations of the study.

We thank the reviewer for acknowledging the value of our study. We believe that we have addressed the concerns raised by the reviewer in the revised version.

Main points.

1. Clarity in the scope of the study.
The study claims to be an evolutionary analysis, as stated in the title, but the work presented is essentially a brief comparative analysis of striatal snRNA-seq datasets across species. There are no evolution-driven analyses included. The title—and corresponding sections such as the Results and Discussion—should be revised accordingly to more accurately reflect the scope of the study.

We thank the reviewer for this comment. In a strict sense, evolutionary analyses seek to understand when and how a trait (or in this case cell types) has evolved. While our study is also, by design, one to understand how striatal cell types have evolved among diverse species, we acknowledge that we are not powered to make accurate evolutionary conclusions given that we only have 7 species across mammalians. Therefore, we have now revised the manuscript, including the title, to better reflect the comparative nature of our study.

2. Limited use of the transcriptomic data and absence of novel insights.
The analytical framework is heavily focused on compositional differences (i.e., relative abundances of cell types), which, although informative, remains largely descriptive. Additional layers of analysis—such as gene set enrichment, transcription factor activity profiling, or cell–cell interaction inference—would substantially enhance the functional interpretation of the findings and the study's overall contribution to the field.
Along similar lines, one of the main findings highlighted by the authors is the identification of two bat-specific

interneuron subtypes, which is positioned as a central point in the Discussion. However, further analysis of these subtypes beyond the identification of marker genes would be valuable to better characterize their potential functional roles.
Overall, the study does not offer any major conceptual advances.

We thank the reviewer for their suggestions; we agree that additional analyses could and do indeed provide further insight. We have now performed the following analyses:

1. We identified differentially expressed genes (DEG) within primates, and then further identified genes that specifically changed in the human lineage. We then compared the ratio of human-specific DEG among all genes tested for differential gene expression analysis within that cell type to uncover which cell types have relatively more changes in the human lineage. We uncovered that the SPN cell types have more human-specific DEGs than other cell types in both caudate and putamen **(Figure 5)**.

2. We then performed weighted gene correlation network analysis (WGCNA) (Langfelder and Horvath 2008) on human datasets to identify correlated gene modules and performed enrichment analyses to further delineate the modules that are enriched for human-specific DEGs, particularly in the SPNs that have relatively more human-specific DEGs. We further detail GO-term enrichment of these modules and highlight biological processes that are altered in SPNs on the human lineage **(Figure 5)**. We added the following to the revised manuscript:

Differential gene expression analysis reveals stronger human specific change in SPNs than glia

Recent studies found species-specific cell types in striatum but not in cortex (Krienen, Goldman et al. 2020). Expanding on this trend, we identified novel interneuron subtypes in P. discolor (Fig. 4). Although we did not observe differences in cell-type abundances among primates, we asked whether transcriptomic differences were present among primate striatal cell types. Therefore, we performed differential gene expression (DGE) analysis within primates across both tissues and all cell types: D1 SPN, D2 SPN, eSPN, interneurons, MOL, OPC, astrocyte, and microglia. We further identified the human-specific differentially expressed genes (HS DEGs) for each cell type (Methods). We found that, overall, there were more HS up and downregulated genes among D1 and D2 SPNs compared to other cell types (Fig. 5C). To statistically test this, we performed chi-square test for the proportions of HS DEGs to all genes tested within that cell type between neurons and glia (Methods) which revealed significantly more HS DEGs in SPNs than glia regardless of the brain regions (Fig. 5D).

To gain more insight into human specific changes in SPNs, we performed WGCNA and found the gene modules for each cell type in human (Methods). We uncovered that the blue and red modules represent the genes that are highly correlated with the previously identified HS upregulated DEGs in SPNs (Fig. 5E). The GO-term enrichment analysis of these modules (Methods) revealed neurotransmitter related activity in blue module genes whereas it revealed synapse related processes for red module genes (Table S10, Fig. 5F). A closer look on these modules yielded several important genes implicated in human disease: genetic ablation of trophoblast glycoprotein, Tpbg, in mice showed motor impairments reminiscent of Parkinson's Disease symptoms (Bossers, Meerhoff et al. 2009, Park, Yoo et al. 2021); Hrh3, is a histamine receptor involved in pathophysiology of a mouse model of a tic disease (Rapanelli, Frick et al. 2017, Rapanelli, Frick et al. 2018), mutations of UNC80 are involved in regulation of neuron electric signals, and cause dyskinesia, developmental delay and/or severe intellectual disability (Perez, Kadir et al. 2016, Wie, Bharthur et al. 2020) (Fig. 5G). Overall, these results indicate an excess of human-specific gene regulation in SPNs compared to other cell types in our dataset and demonstrate the importance of these genes in brain function and disease risk.

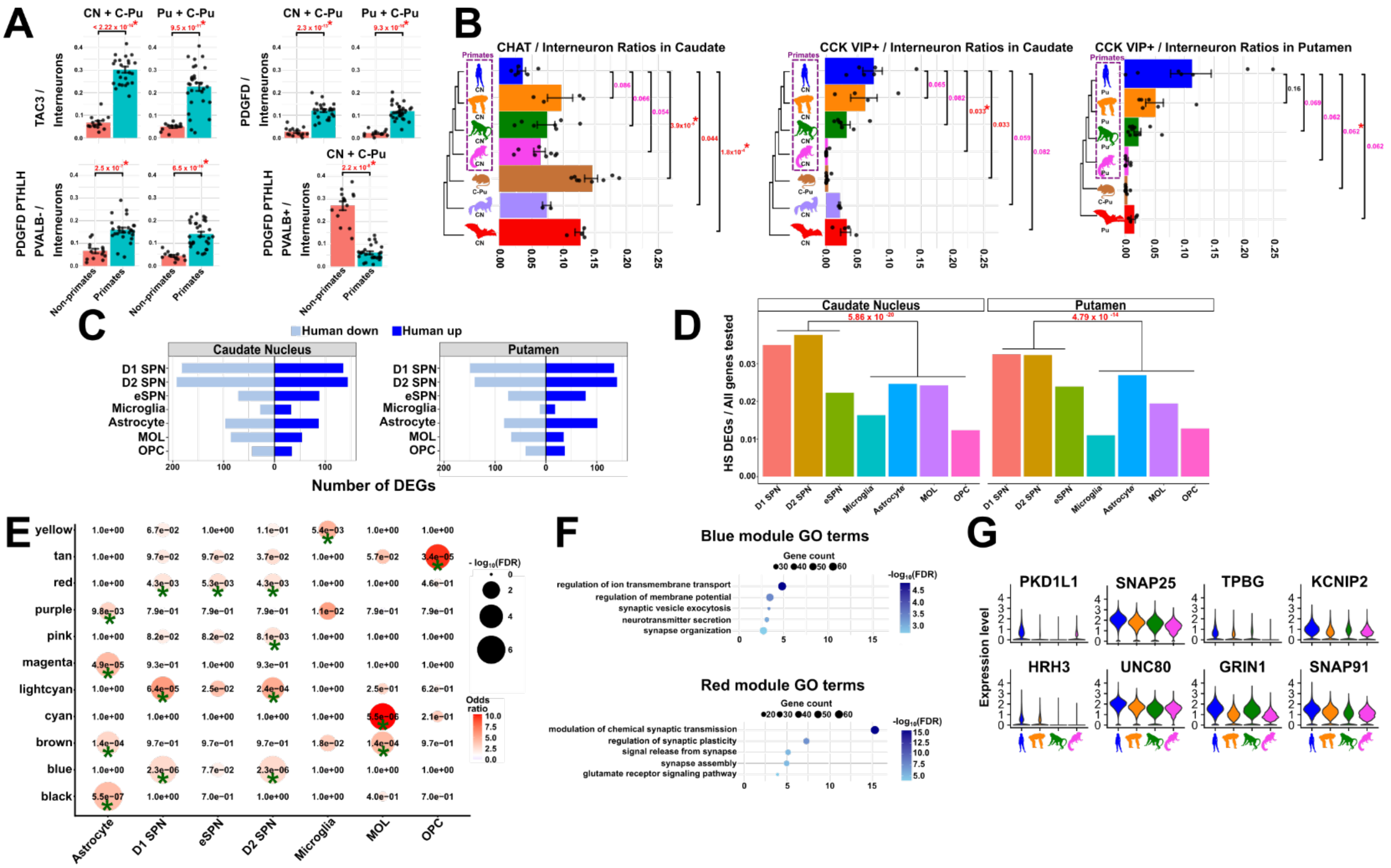

Fig. 5: Comparative analysis reveals human specific changes across striatal interneurons. A. Bar plots showing the ratio of the number of TAC3, PDGFD, PDGFD PTHLH PVALB-, and PDGFD PTHLH PVALB+ interneurons to the number of all interneurons across primate and non-primate CN, Pu, and C-Pu. B. Bar plots showing the ratio of the number of CHAT, CCK VIP-, CCK VIP+ interneurons to the number of all interneurons across mouse caudoputamen, ferret CN, and bat, human, chimpanzee, rhesus macaque, and marmoset CN and Pu. Both in A and B, two tailed t-test was used and nominal p values were multi-test corrected using Benjamini-Hochberg method to assess statistical significance between the groups (FDR < 0.05 is considered significant and written in red; FDR < 0.1 written in pink). Additionally, red asterisk indicates that the comparison is statistically significant in scCODA analysis. Error bars indicate mean ± standard error of the mean. C. Number of human specific differentially expressed genes (HS DEGs) across cell types. D. Ratios of HS DEGs to all genes tested for that cell type. Chi-square test was performed to assess significance in ratios between SPNs (D1 SPN, D2 SPN, and eSPN) and glia (MOL, OPC, Astrocyte, Microglia). (FDR < 0.05 is considered significant and written in red). E. Bubble plot depicting the enrichment of gene modules with HS DEGs per cell type. F. The top significantly enriched gene ontology terms of the blue and red modules (upper and lower panel respectively) (FDR < 0.01). G. Violin plots showing the log normalized expression of the highly variable HS DEGs across species. CN: Caudate Nucleus, Pu: Putamen, C-Pu: Caudoputamen.

3. We further used the WGCNA approach to identify genes modules that are associated with the interneuron cell types found only in bat putamen and highlight several hub genes important for these cell types as well as GO-term enrichment analysis results of these modules **(Figure 4H-J, Table S7)**. We made the following changes to the revised manuscript:

   To gain more insight into bat specific interneurons, we performed weighted gene correlation network analysis (WGCNA) (Langfelder and Horvath 2008) across aggregated and normalized gene expression profiles of all bat interneuron cell types (Methods). We identified blue and cyan modules that were highly enriched for significantly upregulated genes of LMO3 (BAT) and FOXP2 TSHZ2 (BAT) interneurons, respectively (Fig. 4H-J). To identify active biological processes defining these modules, we performed Gene Ontology (GO) enrichment analysis. This analysis revealed significant enrichment primarily for dendrite- and axon-related processes, which were the only terms consistently identified

(Table S7). The lack of more specific GO term enrichment likely reflects the limited current understanding of the functional roles of neurons in this brain region.

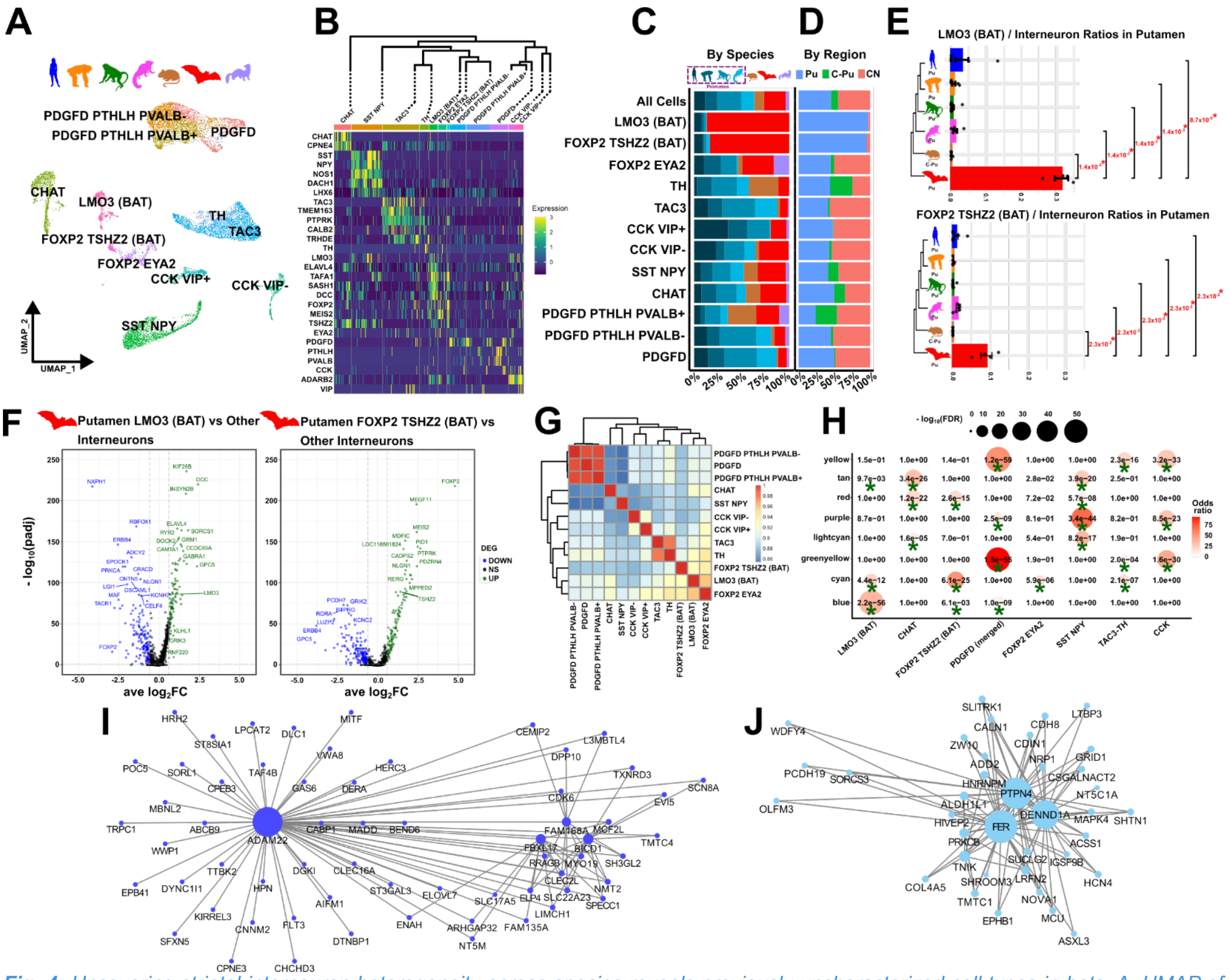

Fig. 4: Uncovering striatal interneuron heterogeneity across species reveals previously uncharacterized cell types in bats. A. UMAP of interneurons integrated across human, chimpanzee, rhesus macaque, marmoset, mouse, bat, and ferret. B. Heatmap showing the scaled (z-score) gene expression of interneuron cell type markers across the interneuron cell types of all the species. Dendogram shows the hierarchical clustering of the interneuron cell types of all the species. C. Bar plot depicting the proportional compositions of the species across interneuron subtypes. D. Bar plot depicting the proportional compositions of the regions across interneuron subtypes. Caudate Nucleus (CN), putamen (Pu), and caudoputamen (C-Pu). E. Bar plots showing the ratio of the number of LMO3 (BAT) and FOXP2 TSHZ2 (BAT) interneurons to the number of all interneurons across mouse C-Pu and bat, human, chimpanzee, rhesus macaque, and marmoset Pu. Two tailed t-test was used and nominal p values were multi-test corrected using Benjamini-Hochberg method to assess statistical significance between the groups (FDR < 0.05 is considered significant and written in red). Additionally, red asterisk indicates that the comparison is statistically significant in scCODA analysis. Error bars indicate mean ± standard error of the mean. F. Volcano plots showing the upregulated and downregulated genes of LMO3 (BAT) and FOXP2 TSHZ2 (BAT) interneurons compared to interneurons within bat Pu. Green dots represent the significantly upregulated genes (Bonferroni-adjusted P < 0.05 & log2FC > 0.6) and blue dots represent the significantly downregulated genes (Bonferroni-adjusted P < 0.05 & log2FC < -0.6). G. Heatmap showing the correlation of normalized gene expressions across interneurons in bat Pu. Dendrogram depicts the hierarchical clustering of interneurons within bat Pu. H. Bubble chart depicting enrichment between each gene module and upregulated DEGs (log2FC = 0.5, FDR < 0.01) in each interneuron subtype. Green asterisk indicates significance of the overlap in Fisher's exact test (FDR < 0.01 and Odds ratio > 2). I,J. Representative visualizations of top 200 connections in blue module and cyan module.

We appreciate the reviewer's other analysis suggestions and fully agree that many additional valuable analyses could be performed with this rich dataset. However, we carefully prioritized analyses that we consider most critical to addressing the central questions of the study. To maintain clarity and focus, we therefore decided not to include further results within the scope of our study. Additionally, we are limited with the current knowledge about discovering the potential functions of these newly found interneuron subtypes.

We also would like the opportunity to highlight what we believe are the major conceptual advances of our study. The discovery of an interneuron cell type in bat putamen alone is a major advance that was not expected or incremental. The presence or absence of an entire cell population among mammalians is rarely observed and might ultimately be linked to either major anatomical or behavioral differences between species. We therefore believe that our findings are uniquely positioned to fuel future research into discovery of clade-specific cell types in mammalian brains as well as understanding the functional significance of cell types potentially restricted to only certain mammalian species.

3. Limited sample size and inconsistent statistical analysis.
Although the integrated dataset spans several species, the number of biological replicates per species and region is limited for some of them (e.g., n = 4 for bat caudate nucleus). This constraint significantly reduces statistical power and makes many comparisons susceptible to noise.

We thank the reviewer for pointing out that our strategy requires further clarification and statistical rigor. We have now provided more detail in our statistical analyses and revised them to incorporate the reviewer's suggestions. With respect to the number samples per species, we are clearly limited by ethical and research guidelines for obtaining these samples. Postmortem tissues from human donors are more accessible than from all other primates. Moreover, the use of bats in research is tightly restricted in Europe and provided by Dr. Vernes in the UK. The bat samples were ones that were collected over several years of tightly monitored research. Additionally, we would also like to point out that many comparative single-cell genomics studies in the recent years have been performed with n=4 per species (Ma, Skarica et al. 2022, Caglayan, Ayhan et al. 2023, Jorstad, Song et al. 2023), both due to limitations in sample availability and the cost of performing single-cell genomics.

Another key limitation of the manuscript lies in the statistical framework used to support its central comparative claims. Several conclusions—such as differences in neuronal and glial proportions or eSPN/SPN ratios across species—are derived from pairwise two-tailed t-tests, without clear description of how values were aggregated (e.g., per individual vs. per cell), whether data were normalized, or whether corrections for multiple testing were applied. Notably, ratio-based comparisons across species (e.g., eSPN/SPN and neuron/glia ratios) require normalization of input cell counts (e.g., controlling for total recovered cells per sample or tissue) to avoid biases introduced by differing cell recovery efficiencies across species or experimental batches. The absence or insufficient description of such normalization undermines the validity of these cross-species comparisons.

We have calculated cell type proportions by dividing the total number of cells per cell type per individual to the total number of cells from the same individual, thus effectively accounting for the differences in cell recovery efficiency in different batches. We have updated our Methods section as follows:

We have calculated cell type proportions by dividing the total number of cells per cell type per individual to the total number of cells from the same individual, thus effectively accounting for the differences in cell recovery efficiency in different batches. We compared the means of these sample-level ratios across species/tissues using the compare_means function in R to perform t-tests. The nominal p values were adjusted for multiple comparisons using the Benjamini-Hochberg false discovery rate (FDR) correction with FDR ≤ 0.05

threshold as well as scCODA (Buttner, Ostner et al. 2021), where an FDR threshold of ≤ 0.1 was applied. Comparisons that achieved significance in both tests, considered statistically significant.

Additionally, the manuscript repeatedly applies uncorrected t-tests across multiple cell types and comparisons (e.g., Table S4; Figs. 2A, 3C–D), increasing the risk of false positives. Correction for multiple testing, such as using the Benjamini-Hochberg false discovery rate (FDR), should be implemented consistently to ensure statistical validity.

More robust compositional analysis methods—such as scCODA—are applied later in the manuscript, but inconsistently. For example, while scCODA is used for some analyses of SPN subtypes, it is not used to validate key comparisons such as neuronal/glial proportions or eSPN/SPN ratios, where its use would be most appropriate given the compositional nature of single-nucleus RNA-seq data. Importantly, in several cases, findings that are significant by t-test lose significance when tested using scCODA, highlighting the need for methodological consistency.

As the reviewer pointed out, for neuron compositional analyses, in addition to t-tests, we also utilized scCODA for all the comparisons. When the significance could not be recapitulated by either of the methods, we have now amended to text to indicate that there is a trend but not significance in the main text:

We tested the statistical significance of the comparisons using both a two tailed t-test and a more robust proportional analysis of single cell-data (scCODA) that correctly accounts for the assumptions imposed by the nature of the proportional data (Buttner, Ostner et al. 2021). While neuronal proportions were similar between human-chimpanzee, macaque and marmoset neuronal proportions were greater than human with either statistical significance or a strong trend (Table S2 and Fig. 2A). As an outgroup to the primates, we calculated neuronal proportions in mouse and found that they were significantly higher than humans (two-tailed t-test, FDR = 4.8 x 10-9 and scCODA = significant; Table S2 and Fig. 2A).

Similarly, we sought to understand whether the neuronal proportion difference between CN and Pu (Fig. 2D) might be driven by proportional changes in glial cell types. We observed that CN had significantly different neuron-to-OPC ratios compared to Pu only in bats (two-tailed t-test, FDR = 2.1 x 10-3 and scCODA = significant; Fig. S2E-F) and strong trends in other glial cell types (Fig. S2E–H). To further assess neuron-to-MOL proportions between bat CN and Pu with an orthogonal method, we performed smFISH, which confirmed a significant difference in the neuron-to-MOL ratio (p = 0.0133, Methods; Fig. 2F-I). Interestingly, the difference between bat CN and bat Pu was especially low in astrocytes with only a ~1.5-fold increase of neuron / astrocyte in CN compared to at least 2-fold increase in all other glial cell types (Fig. S2E-H).

We believe the reviewer missed this, as in the previous version of the manuscript, we already provided the scCODA results for eSPN/SPN ratios along with odds ratios in Fig 3C-D and Table S3.

In summary, the inconsistent application of compositional methods, lack of clear normalization procedures in ratio-based analyses, and absence of multiple testing corrections collectively reduce the statistical rigor and interpretability of the results.

We have now consistently applied reviewer's suggestions to all applicable analyses throughout the study and added the following to Methods:

We have calculated cell type proportions by dividing the total number of cells per cell type per individual to the total number of cells from the same individual, thus effectively accounting for the differences in cell

recovery efficiency in different batches. We compared the means of these sample-level ratios across species/tissues using the compare_means function in R to perform t-tests. The nominal p values were adjusted for multiple comparisons using the Benjamini-Hochberg false discovery rate (FDR) correction with FDR ≤ 0.05 threshold as well as scCODA (Buttner, Ostner et al. 2021), where an FDR threshold of ≤ 0.1 was applied. Comparisons that achieved significance in both tests, considered statistically significant.

Minor Points.

1. Figure Clarity and Formatting.
-Standardize axis scales across comparable plots (e.g., Figs. 3C–D, 4E–F, 4H–I, S4D–E) to facilitate accurate visual comparisons.

We have now standardized the axis scales across the indicated comparable plots, with the exception of Figs. S4D–E. In Fig. S4D, the data points are already densely clustered, and applying a standardized scale would substantially reduce readability. Moreover, we do not make any direct comparative claims between panels S4D and S4E.

-Maintain consistent species color schemes across figures (e.g., between Fig. 4A and 4C) to enhance interpretability.

In Fig. 4C, we chose to show the primates as shades of blue to compare the primate vs non-primate compositions as well as individual species comparisons by looking at the same plot.

-Improve layout alignment and spacing in multi-panel figures (e.g., Fig. 1C).

We have improved the alignment and spacing in Fig 1.

-Correct residual graphical artifacts (e.g., square-like borders, contour lines, "=" lines) in Supplementary Figures (e.g., S1C, S1D, S2A, S2D, S2H), likely due to vector-to-raster format conversions.

We have corrected the residual artifacts in Fig S1 and Fig S2.

-Clarify whether expression heatmaps and figures showing negative values (e.g., Fig. 4B) reflect log, z-score, or other transformations; specify this in the figure legends and Methods. (Negative values are unexpected following standard normalization procedures commonly applied in sc/sn RNA-seq data processing)

We have added the z-score explanation in each heatmap figure legend and Methods.

-Supplementary Figure 1D lacks clarity, and the gene names are not readable.

We have improved the graphics of the text in Fig S1D.

2. Statistical Annotation and Reporting.
-Specify the summary statistic used in figures showing aggregated expression as it is the case of violin plots (e.g., mean vs. median in Fig. 1C).

Figure 1C: We utilized Seurat VlnPlot function to generate the figure, there were no summary statistics used. Violin plots display the distribution of log normalized expression levels for a given gene across individual nuclei. Instead of plotting individual data points (dots), the density of expression values is represented as the

shape of the violin. Further addition of mean/median to the figure would reduce the readability, as the figure is already very small. Therefore, we decided to leave it as is.

-Report numerical values with appropriate statistical summaries (e.g., mean ± SEM or SD) when describing observed trends (e.g., Fig. 2A).

We have decided to remove this part in the main text as we talk about the caudate and putamen differences in the next paragraph:

…although bat neuronal proportions displayed high variability across striatal regions, reflecting marked differences between CN and Pu (mean ± SD = 59.5% ± 20.3%) (Table S2 and Fig. 2A-C).

-For correlation analyses (e.g., Figs. 4G), report correlation coefficients (r) and associated p-values to support conclusions.

We have now reported correlation coefficients in the main text. Due to the large number of genes used to compute correlations (n ≈ 19,600), all pairwise Pearson correlations were highly significant (adjusted p = 0, as the values were below R's numerical reporting threshold). We therefore focus on the magnitude of correlation coefficients rather than p-values:

The correlation analysis showed that LMO3 interneurons were most similar to FOXP2 EYA2 and FOXP2 TSHZ2 (BAT) interneurons (r = 0.96 and 0.93 respectively, Fig. 4G). The FOXP2 EYA2-FOXP2 TSHZ2 (BAT)-LMO3 (BAT) clade was most similar to the TH-TAC3 interneuron clade, indicating that these cells may have similar functions in bat Pu and validating the previous findings with all the species and tissues (r = 0.90–0.94, Fig. 4B and G).

3. Validation and Statistical Claims.
-In smFISH validation (e.g., Figs. 2H–I), clearly describe the analysis in the Methods section, including the number of biological replicates and exact statistical tests used.

We now provide more details about smFISH validation strategy in Methods:

Cell numbers (MOG+, RBFOX3+, etc.) were quantified manually in double blind manner using Fiji. For example, MOG was defined as double positive cells for MOG and DAPI, RBFOX3 was defined as double positive cells for RBFOX3 and DAPI. To compare neuronal and oligodendrocyte composition in the caudate between humans (n = 4) and bats (n = 4), between the caudate (n = 4) and putamen (n = 4) in bats, and to assess the eSPN/SPN ratio between humans (n = 2) and mice (n = 3), we obtained at least two measurements per individual (Table S1). We quantified CASZ1+/DRD1+ cells (eSPNs) and DRD1+ or DRD2+ cells (SPNs) in the caudate (human) and caudate - putamen (mouse). Data were analyzed using a linear mixed model with species as the fixed factor and individual as the random factor (lme4 package in R, with REML = FALSE). Statistical significance was set at FDR $p < 0.05$.

-Avoid claiming "significant" differences (e.g., in Fig. 4I, human vs. chimpanzee) when statistical significance is not achieved; instead, refer to such results as trends and provide actual p-values.

We have made this change.

-Please avoid phrasing such as "the algorithm considered it not significant." Instead, simply state whether the result is significant or not, along with the p-value and the test statistic.

We have made this change.

4. Compositional Analyses.
Justify the choice of iSPNs as the reference population in scCODA analyses; ideally, add a supplementary figure showing their relative stability across samples and species.

We already provide the stable distributions of iSPNs (D2 SPNs) across samples and species in stacked bar plots, Fig. S3C-D. We have now added the total dispersion- abundance plot of D2 SPNs in Fig. S3E.

5. Quality Control and Data Processing.
-Clarify whether the nuclear quality control (e.g., intronic read thresholds as shown in Fig. S1B) was the only QC step performed. If additional filters (e.g., mitochondrial gene content, total gene counts, or doublet detection) were used, these should be explicitly described.

In addition to providing all the QC scripts on our Github page, we have now added further details about QC in the Methods:

For each sample (individual), we normalized gene counts using log normalization, found variable genes necessary to generate principal components using FindVariableFeatures (Stuart, Butler et al. 2019). We then selected the top 2000 genes that are variable across samples using SelectIntegrationFeatures with default parameters (Stuart, Butler et al. 2019). We generated z-scores of these genes using ScaleData then integrated samples within each species using FindIntegrationAnchors with normalization.method = 'LogNormalize' and reduction method rcpa and IntegrateData with k.weight = 30, normalization.method = 'LogNormalize' (Stuart, Butler et al. 2019). We then generated principal components using RunPCA with default parameters and clustered cells using FindNeighbors with SNN and FindClusters with Louvain algorithm (resolution 1) and visualized using RunUMAP (dims = 1:20 and reduction = 'pca') and DimPlot (Stuart, Butler et al. 2019). We removed clusters with low intronic read ratios (< 0.5), we also removed clusters with an unusually high number of detected genes accompanied with a high level of expression of at least two typically distinct marker genes as potential nuclear doublets. In addition to doublets, we also removed non-cells (empty), endothelial cells, and pericytes (FLT1, DUSP1, EBF1, PDGFRB expressing clusters) from the dataset. We re-clustered the nuclei and repeated this process if needed until no such clusters were found.

-Detail the clustering methodology, including the algorithm (e.g., Louvain or Leiden), resolution used, and total number of clusters generated.

We added the following in the Methods:

…and clustered cells using FindNeighbors with SNN and FindClusters with Louvain algorithm (resolution 1) and visualized using RunUMAP (dims = 1:20 and reduction = 'pca') and DimPlot (Stuart, Butler et al. 2019).

-Report how many clusters were excluded based on QC (e.g., low intronic ratios, high gene counts, or expression of non-neuronal markers like FLT1, DUSP1, EBF1, PDGFRB).

During QC, our cluster filtering strategy relies on an iterative process of clustering – filtering – reclustering until all clusters supported the QC standards we provide in the Methods (mean intronic read ratio > 0.5, no doublets, no empty clusters). Therefore, it would not be meaningful to provide the number of clusters excluded. Instead, we provide the number of nuclei before and after filtering in the main text:

After stringent quality control, we identified 156,154 cells in human (pre filter = 185,531), 152,404 cells in chimpanzee (pre filter = 122,274), 156,178 cells in rhesus macaque (pre filter = 163,025), 91,378 cells in marmoset (pre filter = 109,913), 78,623 cells in mouse (pre filter = 91,054), and 120,742 cells in bat (pre filter = 123,362) (Fig. 1).

-Specify the normalization procedure applied (e.g., log-normalization, SCTransform); if no normalization was performed, this should be justified.

Answered above.

Reviewer #1 (Remarks on code availability):

I have reviewed the code, which includes a README file and the scripts used for the analysis; however, I have not executed it.

Reviewer #2 (Remarks to the Author):

In the present study Buyukkahraman et al. study the evolution of neuron types in the dorsal striatum. They used data from four primates - human, chimpanzee, macaque, and marmoset, and three nonprimate species - mice, bats, and ferrets. They found that primates had a lower neuron to glia ratio than non-primates, that primate DS contained a larger fraction of eSPNs, and they revealed some new interneuron types in the bat. The data appear to be analyzed properly, I liked the use of the liftover tool across species. That primate DS contains a larger fraction of eSPNs and the novel bat interneuron populations could constitute significant contributions. Still I have a couple of questions and concerns.

Major Concerns
The authors need to provide more details about the dissections. The drawing in figure 1 appears to indicate that human samples were taken from the rostral striatum, the He, Kleyman 2021 data they used is also rostral striatum, but the bat sampling shown in S1 takes cells broadly along the rostral-caudal extent. Can the 'novel' cell types in the bat be explained by the fact that they sampled more broadly? Moreover, these are the only species I could find any information for. The striatum is not a homogenous lump. More info needed.

We understand the reviewer's concern and apologize that the schematic in previous Figure 1 was misleading. We have direct images of the bat tissue sampling as indicated by the reviewer. For the samples from nonhuman primates that our team generated, we were consistent in sampling brains from coronal slabs at the level of the rostral striatum, where the caudate and putamen are both prominent and the internal capsule has begun to separate them. It is anterior to the appearance of the globus pallidus and thalamus. Please see below for an image of this level from a rhesus atlas for reference.

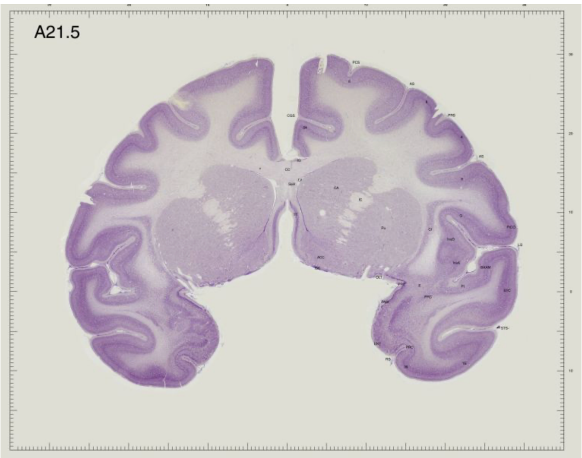

While we cannot be certain of the location of the sampling of the publicly available data, the overall correspondence among the other primate samples gives us confidence in their location. Finally, the human samples were obtained from the NIH Neurobiobank, specifically the collection of Mt. Sinai. We reached out to this brain bank personnel and they responded that they do not have photographic images. Again, however, due to the overall correspondence of our cell type data among the primates, we have strong confidence in the overall similarity among species. In addition, we used independent human samples to carry out our validation smFISH studies, underscoring the reproducibility of our data across new dorsal striatum tissue samples. Nonetheless, to the reviewer's point below, we add the caveat that we cannot be 100% certain that we are comparing the exact same functional regions within the dorsal striatum.

As to the reviewer's concern whether the bat-specific interneuron subtypes might be contributed from more caudal portions of the putamen, we find it unlikely that such cell types would be completely absent in the putamen of 6 different animals, especially from the entire caudoputamen of mouse. Given the vast variation in brain sizes and potential lack of uniformity in the expansion/shrinkage of different brain regions during evolution, addressing such a concern in full would require counting rare interneuron cell types section by section in the putamen of different species. Furthermore, given the rarity of this cell type, it would also require a highly sensitive method such as spatial transcriptomics at single-cell resolution to achieve a definitive result. Such a comprehensive undertaking may be achieved in future studies, but it remains outside the means and scope of our current study.

A point somewhat related to my first concern is that the authors appear to give no consideration of functional differences between the species that could account for cell type differences. Primates have a huge caudate body because they have large associative and oculomotor cortical territories that are absent in other species. Bats, I imagine, have unique striatal regions dedicated to hearing. This point is similar to point one in that we need to ensure we are comparing apples to apples. However, it is slightly more subtle because, in an ideal world, we would only compare functionally homologous regions of the striatum. If that is not possible, then the authors should be more cautious with their claims.

We thank the reviewer for this point and now add the following text to our discussion:

While we have observed strong transcriptional conservation of the majority of cell types across species in either the caudate or putamen, as might be expected, a caveat to our study is that we cannot be completely certain that we are comparing functionally homologous regions. Future studies that manipulate cell types in model systems, such as the marmoset or ferret, may ultimately validate the direct cell type comparisons.

The authors refer to dSPNs and iSPNs, but they don't show that the SPNs are actually part of the direct (d)

and indirect (i) pathways. These authors are not the only ones to do this, but it still isn't accurate. Of course, most DRD1-expressing SPNs are part of the direct pathway, but there are some DRD1 neurons that project to GPe. I suggest that without data on the actual projection targets, we should annotate cell types with gene markers rather than assumptions.

We thank the reviewer for this suggestion and have now changed the labels to "D1 SPNs" and "D2 SPNs".

Reviewer #3 (Remarks to the Author):

I co-reviewed this manuscript with one of the reviewers who provided the listed reports. This is part of the Nature Communications initiative to facilitate training in peer review and to provide appropriate recognition for Early Career Researchers who co-review manuscripts.

References

Bossers, K., G. Meerhoff, R. Balesar, J. W. van Dongen, C. G. Kruse, D. F. Swaab and J. Verhaagen (2009). "Analysis of gene expression in Parkinson's disease: possible involvement of neurotrophic support and axon guidance in dopaminergic cell death." Brain Pathol **19**(1): 91-107.

Buttner, M., J. Ostner, C. L. Muller, F. J. Theis and B. Schubert (2021). "scCODA is a Bayesian model for compositional single-cell data analysis." Nat Commun **12**(1): 6876.

Caglayan, E., F. Ayhan, Y. Liu, R. M. Vollmer, E. Oh, C. C. Sherwood, T. M. Preuss, S. V. Yi and G. Konopka (2023). "Molecular features driving cellular complexity of human brain evolution." Nature **620**(7972): 145-153.

Jorstad, N. L., J. H. T. Song, D. Exposito-Alonso, H. Suresh, N. Castro-Pacheco, F. M. Krienen, A. M. Yanny, J. Close, E. Gelfand, B. Long, S. C. Seeman, K. J. Travaglini, S. Basu, M. Beaudin, D. Bertagnolli, M. Crow, S. L. Ding, J. Eggermont, A. Glandon, J. Goldy, K. Kiick, T. Kroes, D. McMillen, T. Pham, C. Rimorin, K. Siletti, S. Somasundaram, M. Tieu, A. Torkelson, G. Feng, W. D. Hopkins, T. Hollt, C. D. Keene, S. Linnarsson, S. A. McCarroll, B. P. Lelieveldt, C. C. Sherwood, K. Smith, C. A. Walsh, A. Dobin, J. Gillis, E. S. Lein, R. D. Hodge and T. E. Bakken (2023). "Comparative transcriptomics reveals human-specific cortical features." Science **382**(6667): eade9516.

Krienen, F. M., M. Goldman, Q. Zhang, C. H. D. R. R, M. Florio, R. Machold, A. Saunders, K. Levandowski, H. Zaniewski, B. Schuman, C. Wu, A. Lutservitz, C. D. Mullally, N. Reed, E. Bien, L. Bortolin, M. Fernandez-Otero, J. D. Lin, A. Wysoker, J. Nemesh, D. Kulp, M. Burns, V. Tkachev, R. Smith, C. A. Walsh, J. Dimidschstein, B. Rudy, S. K. L, S. Berretta, G. Fishell, G. Feng and S. A. McCarroll (2020). "Innovations present in the primate interneuron repertoire." Nature **586**(7828): 262-269.

Langfelder, P. and S. Horvath (2008). "WGCNA: an R package for weighted correlation network analysis." BMC Bioinformatics **9**: 559.

Ma, S., M. Skarica, Q. Li, C. Xu, R. D. Risgaard, A. T. N. Tebbenkamp, X. Mato-Blanco, R. Kovner, Z. Krsnik, X. de Martin, V. Luria, X. Marti-Perez, D. Liang, A. Karger, D. K. Schmidt, Z. Gomez-Sanchez, C. Qi, K. T. Gobeske, S. Pochareddy, A. Debnath, C. J. Hottman, J. Spurrier, L. Teo, A. G. Boghdadi, J. Homman-Ludiye, J. J. Ely, E. W. Daadi, D. Mi, M. Daadi, O. Marin, P. R. Hof, M. R. Rasin, J. Bourne, C. C. Sherwood, G. Santpere, M. J. Girgenti, S. M. Strittmatter, A. M. M. Sousa and N. Sestan (2022). "Molecular and cellular evolution of the primate dorsolateral prefrontal cortex." Science **377**(6614): eabo7257.

Park, S., J. E. Yoo, G. B. Yeon, J. H. Kim, J. S. Lee, S. K. Choi, Y. G. Hwang, C. W. Park, M. S. Cho, J. Kim, D. Na, H. W. Kim, D. S. Kim and D. W. Kim (2021). "Trophoblast glycoprotein is a new candidate gene for Parkinson's disease." NPJ Parkinsons Dis **7**(1): 110.

Perez, Y., R. Kadir, M. Volodarsky, I. Noyman, H. Flusser, Z. Shorer, L. Gradstein, R. Y. Birnbaum and O. S. Birk (2016). "UNC80 mutation causes a syndrome of hypotonia, severe intellectual disability, dyskinesia and dysmorphism, similar to that caused by mutations in its interacting cation channel NALCN." J Med Genet **53**(6): 397-402.

Rapanelli, M., L. Frick, K. Jindachomthong, J. Xu, H. Ohtsu, A. C. Nairn and C. Pittenger (2018). "Striatal Signaling Regulated by the H3R Histamine Receptor in a Mouse Model of tic Pathophysiology." Neuroscience **392**: 172-179.

Rapanelli, M., L. Frick, V. Pogorelov, H. Ohtsu, H. Bito and C. Pittenger (2017). "Histamine H3R receptor activation in the dorsal striatum triggers stereotypies in a mouse model of tic disorders." Transl Psychiatry **7**(1): e1013.

Wie, J., A. Bharthur, M. Wolfgang, V. Narayanan, K. Ramsey, C. R. R. Group, K. Aranda, Q. Zhang, Y. Zhou and D. Ren (2020). "Intellectual disability-associated UNC80 mutations reveal inter-subunit interaction and dendritic function of the NALCN channel complex." Nat Commun **11**(1): 3351.

We thank the reviewers for their sincere and helpful comments and address their remaining concerns point by point below.

Reviewer #1 (Remarks to the Author):

Thank you to the authors for the careful and detailed revision. The manuscript has improved, particularly in the methodological description, statistical clarification, and additional downstream analyses. The datasets generated—especially the new insights in bat —represent a valuable contribution that will be of interest to the community.
That said, after evaluating the revised manuscript and responses, I do not consider the work reaches the level of conceptual advance expected for publication in Nature Communications.

The authors have made a genuine effort to expand the analytical depth, including additional differential expression and WGCNA analyses for both the human lineage and the bat-specific interneurons. However, in my assessment, these additions remain largely descriptive and do not substantially elevate the central biological insight of the study.

Regarding the bat findings, the identification of putamen-specific interneuron subtypes is interesting and potentially important. The additional module analysis provides some characterization, but the functional interpretation remains limited, and the authors appropriately note the current knowledge gaps. Consequently the study remains short of delivering a strong mechanistic.

The authors have also improved the statistical framework and clarified normalization and multiple-testing procedures. These revisions address several of the prior technical concerns. However, even with these improvements, the overall advance of the manuscript remains primarily descriptive rather than conceptual.
In summary, I believe the datasets presented here will be a useful resource but in its current form the manuscript does not yet provide the level of conceptual insight and integrative interpretation expected for Nature Communications.
I hope these comments are helpful to the authors in further developing their work.

We thank the reviewer for their thoughtful and constructive comments on our revision process. We are encouraged that the reviewer recognizes the improvements in methodological clarity, statistical rigor, and the addition of downstream analyses, as well as the value of the datasets generated, particularly for the bat.

We respectfully disagree with the reviewer's assessment regarding the level of conceptual advance. Following the reviewer's suggestions, we have substantially expanded the analytical framework, including additional differential expression and WGCNA analyses across both the human lineage and bat-specific interneurons. These analyses were not intended solely to increase descriptive depth, but rather to uncover conserved and lineage-specific regulatory programs that provide new biological insights into interneuron diversity and evolution.

At the same time, we acknowledge the reviewer's point that parts of the study remain descriptive in nature, particularly in the context of large-scale comparative transcriptomic analyses. However, we

believe that the integration of cross-species datasets, combined with cell type–specific regulatory and evolutionary insights, moves beyond a purely descriptive resource and contributes meaningful conceptual advances to the field such as discovery of previously uncharacterized cell types in *P. discolor.*

Importantly, studies with a similar combination of large-scale dataset generation and integrative comparative analysis have been published in *Nature Communications*, where the value lies in both the resource and the biological insights derived from it (Avican, Aldahdooh et al. 2021, Bo, Li et al. 2023, Simpson, Strange et al. 2024). In this context, we believe our study is well aligned with the scope and expectations of the journal.

A major remaining concern is the disconnect between the Results and the Discussion. While the Results now build a coherent narrative—from broad cellular proportions to human-specific transcriptomic changes (Fig. 5)—the Discussion does not meaningfully engage with these final molecular findings. In particular, the reported enrichment of human-specific DEGs in SPNs is not interpreted in a way that clarifies its functional or evolutionary significance. Likewise, although several highlighted genes (e.g., Tpbg, Hrh3, UNC80) are linked to neurological conditions, the manuscript does not adequately discuss what it might biologically imply that these genes show human-specific upregulation. As written, the conceptual implications of these analyses remain unclear.

We thank the reviewer for their comments and agree with the reviewer. We added the following to our Discussion:

We further found that spiny projection neurons (SPNs), the principal neuronal population of the striatum, exhibit the strongest human-specific transcriptional divergence among all cell types, with enriched changes in genes related to synaptic function and neurotransmitter signaling, many of which are also implicated in neurological disease, underscoring their potential importance in human-specific brain function and vulnerability.

Reviewer #1 (Remarks on code availability):

The code was reviewed but not executed

Reviewer #2 (Remarks to the Author):

The authors have addressed my concerns.

Reviewer #3 (Remarks to the Author):

I co-reviewed this manuscript with one of the reviewers who provided the listed reports. This is part of the Nature Communications initiative to facilitate training in peer review and to provide appropriate recognition for Early Career Researchers who co-review manuscripts.

References

Avican, K., J. Aldahdooh, M. Togninalli, A. Mahmud, J. Tang, K. M. Borgwardt, M. Rhen and M. Fallman (2021). "RNA atlas of human bacterial pathogens uncovers stress dynamics linked to infection." Nat Commun **12**(1): 3282.

Bo, T., J. Li, G. Hu, G. Zhang, W. Wang, Q. Lv, S. Zhao, J. Ma, M. Qin, X. Yao, M. Wang, G. Z. Wang and Z. Wang (2023). "Brain-wide and cell-specific transcriptomic insights into MRI-derived cortical morphology in macaque monkeys." Nat Commun **14**(1): 1499.

Simpson, L., A. Strange, D. Klisch, S. Kraunsoe, T. Azami, D. Goszczynski, T. Le Minh, B. Planells, N. Holmes, F. Sang, S. Henson, M. Loose, J. Nichols and R. Alberio (2024). "A single-cell atlas of pig gastrulation as a resource for comparative embryology." Nat Commun **15**(1): 5210.
